# Delayed feedback embedded in perception-action coordination cycles results in anticipation behavior during synchronized rhythmic action: A dynamical systems approach

**DOI:** 10.1371/journal.pcbi.1007371

**Published:** 2019-10-31

**Authors:** Iran R. Roman, Auriel Washburn, Edward W. Large, Chris Chafe, Takako Fujioka

**Affiliations:** 1 Center for Computer Research in Music and Acoustics, Department of Music, Stanford University, Stanford, United States of America; 2 Stanford Neurosciences Graduate Training Program, Stanford University, Stanford, United States of America; 3 Department of Computer Science and Engineering, University of California San Diego, La Jolla, United States of America; 4 Department of Psychological Sciences, University of Connecticut, Storrs, United States of America; 5 Department of Physics, University of Connecticut, Storrs, United States of America; 6 Wu Tsai Neurosciences Institute, Stanford University, Stanford, United States of America; University of Pittsburgh, UNITED STATES

## Abstract

Dancing and playing music require people to coordinate actions with auditory rhythms. In laboratory perception-action coordination tasks, people are asked to synchronize taps with a metronome. When synchronizing with a metronome, people tend to anticipate stimulus onsets, tapping slightly before the stimulus. The anticipation tendency increases with longer stimulus periods of up to 3500ms, but is less pronounced in trained individuals like musicians compared to non-musicians. Furthermore, external factors influence the timing of tapping. These factors include the presence of auditory feedback from one’s own taps, the presence of a partner performing coordinated joint tapping, and transmission latencies (TLs) between coordinating partners. Phenomena like the anticipation tendency can be explained by delay-coupled systems, which may be inherent to the sensorimotor system during perception-action coordination. Here we tested whether a dynamical systems model based on this hypothesis reproduces observed patterns of human synchronization. We simulated behavior with a model consisting of an oscillator receiving its own delayed activity as input. Three simulation experiments were conducted using previously-published behavioral data from 1) simple tapping, 2) two-person alternating beat-tapping, and 3) two-person alternating rhythm-clapping in the presence of a range of constant auditory TLs. In Experiment 1, our model replicated the larger anticipation observed for longer stimulus intervals and adjusting the amplitude of the delayed feedback reproduced the difference between musicians and non-musicians. In Experiment 2, by connecting two models we replicated the smaller anticipation observed in human joint tapping with bi-directional auditory feedback compared to joint tapping without feedback. In Experiment 3, we varied TLs between two models alternately receiving signals from one another. Results showed reciprocal lags at points of alternation, consistent with behavioral patterns. Overall, our model explains various anticipatory behaviors, and has potential to inform theories of adaptive human synchronization.

## Author summary

When navigating a busy sidewalk, people coordinate their behavior in an orderly manner. Other activities require people to carefully synchronize periodic actions, as in a group rowing or marching. When individuals tap in synchrony with a metronome, their taps tend to anticipate the metronome. Experiments have revealed that factors like musical expertise, the presence of a synchronizing partner, auditory feedback, and the sound travel time, all systematically affect the tendency to anticipate. While researchers have hypothesized a number of potential mechanisms for such anticipatory behavior, none have successfully accounted for all of the effects. Previous research on coupled physical systems has shown that when one system receives input from a second system, plus its own delayed signal as input, this causes system 1 to anticipate system 2. We hypothesize that the tendency to anticipate is the result of delayed communication between neurons. Our work demonstrates the ability of delay-coupled physical systems to capture human anticipation and the effect of external factors in the anticipation tendency. Our model supports the theory that delayed communication within the nervous system is crucial to understanding anticipatory coordinative behavior.

## Introduction

In social settings, people must coordinate actions in order to carry out fundamental activities like walking or talking. Some activities require individuals to precisely time repetitive actions such as dancing, rowing, or music making, resulting in synchronization with external information, shared among a group of individuals. This kind of perception-action coordination is also sometimes called sensorimotor synchronization, because it is considered to depend on communication between the sensory and motor areas of the nervous system [1]. The simplest form of synchronization happens when individuals tap in synchrony with an isochronous stimulus. In doing so, individuals’ actions on average tend to slightly precede the stimulus, resulting in a mean negative asynchrony between the stimulus onsets and corresponding taps. This negative mean asynchrony has been observed consistently in the literature. Anticipation is observed when humans tap with an isochronous stimulus with inter-onset-intervals (IOIs) ranging from 300ms to 4800ms [2]. However, the asynchronies vary widely and can be positive, on average, for an individual tapping with IOIs longer than 2000ms [2]. For IOIs greater than 2000ms, asynchronies may show a bimodal distribution; some taps precede the stimulus while others follow it, with longer IOIs resulting in more taps that follow the stimulus and fewer that precede it [3]. The anticipation tendency is influenced by musical experience, as tap timing in musicians is closer to the stimulus than that in non-musicians for IOIs between 1000ms and 3500ms [4]. When the IOI is greater than 5000ms, more taps occur after the stimulus than before the stimulus, suggesting that people are more reactive upon hearing the next beat [5]. Similarly, when synchronizing with the beat underlying complex surface rhythms (e.g., syncopation), the mean asynchrony shifts to the positive side [6–8] (see [9] for a review). Collectively, these results indicate that the anticipation tendency depends not only the IOI, but also, the expertise level, complexity of the rhythms and the task requirements.

Two or more people can also perform coordinated rhythmic behavior. For example, two musicians may alternate taps to maintain a common stable tempo. To achieve coordination, they must employ an interactive and adaptive strategy and adjust their tap timing based on their own timing as well as their partner's. The asynchrony of one person tends to copy the previous asynchrony produced by their partner [10–11]. This tendency is not observed when one of the partners cannot hear the actions of the other, indicating that the auditory feedback between synchronizing partners can affect their ability to coordinate [12]. It appears that in the presence of auditory feedback, coordination is not affected by the presence or absence of other non-auditory information [10,13]. Another factor that affects auditory information in coordination is transmission latency (TL), which refers to a delay between the time at which an event occurs and the time when the associated auditory information is available. TLs are a result of the transmission of information across a physical distance separating two synchronizing individuals [14]. Analogous latencies can be observed when musicians at remote locations play music together over the internet. One study has examined the effect of the TL between two individuals trying to alternately clap a rhythm [14]. The authors observed that stable synchronization is achieved with small TLs (10-20ms), while musicians collectively speed up for TLs shorter than 10ms, and slow down for latencies longer than 20ms [14].

Different models try to explain how neural computations give rise to anticipation and coordination. Researchers have proposed that the anticipation results from the combination of information from different modalities, based on differences in the axonal distances between the hand, the ear, and the brain [15–16]. For example, the sensory accumulation model [17] proposes that the time difference in central-peripheral signal communications between the auditory and motor systems may be responsible for the anticipation; it takes less time for pacing stimuli to travel from the ear to the auditory cortex compared to the time it takes a signal to travel from the fingertips to the brain and vice versa. As a result, taps precede external auditory signals so somatosensory and auditory information will coincide at the level of the central nervous system. One prediction of the sensory accumulation model is that louder stimuli will result in smaller anticipation. Białuńska, Bella, & Jaśkowski tested this theory behaviorally, and found that the prediction was not confirmed, indicating that other sensorimotor mechanisms must be involved [18]. Another model proposes perceptual underestimation of IOIs [19]. This predicts that anticipation should be reduced when periodic stimuli are subdivided into equidistant strong and weak beats [20–22]. However, due to conflicting results, an integrative and convergent explanation is still yet to be established (see [23] for a review).

Mechanisms underlying generalized anticipatory behavior beyond the simple anticipatory phenomena have recently been of interest. Dubois [24] has identified two main theories as to how anticipatory behavior arises. The first one is called ‘weak anticipation theory’, proposing that anticipation occurs as the result of inferences produced by internal models. Specifically, this theory argues that because the brain generates representations of likely future events based on external information, anticipation is the result of the brain’s eagerness to confirm the correctness of these representations [25–26]. The second perspective is termed ‘strong anticipation theory’, which suggests that anticipation results from the homeostatic coupling of information between an organism and its environment. Stepp and Turvey [27] note that, in its original Latin meaning, anticipation refers to the act of “following a path before.” Accordingly, anticipation must involve not only correctly predicting another model’s actions but also realizing that prediction with one’s own actions [27]. Stepp and Turvey also explained that homeostatic mechanisms can keep an anticipating organism in a proactive state [27]. One can see that a major drawback of the weak anticipation theory is that it cannot explain how some physical systems anticipate, even without the ability to carry out inference (e.g., laser semiconductors and electronic circuits) [28–29]. In contrast, strong anticipation theory can explain anticipation as a theoretical framework that generalizes across physical systems [27]. The present study is aimed at exploring how the strong anticipation theory could further explain various results in rhythmic coordination in an integrative manner, including anticipatory synchronization, by conducting computational simulations.

Specifically, ‘anticipatory synchronization’ in strong anticipation emerges from the coupling between a 'response' system and a 'driver' system (e.g., stimulus input) wherein the response system also receives delayed feedback about its own activity. One of the major strengths of the strong anticipation approach is that it accounts for anticipatory phenomena beyond human behavior, and that collectively all such phenomena can be modeled as coupled dynamical systems [27,28–29]. There are parallels between a dynamical system with delayed feedback and the delayed communication between different areas in the sensorimotor system of humans carrying out synchronization. Synchronization requires communication between auditory, premotor and motor brain areas [30–32], which involves delayed transmission of neural information. Moreover, it has been suggested that these delays inherent within the human sensorimotor system may act like ones in coupled dynamical systems with delayed feedback inputs [28,31–32], supporting the strong anticipation theory. If this holds true, a low-dimensional dynamical systems model could explain anticipation in perception-action coordination.

To simulate periodic perception-action coordination, we use an oscillator described by Eq (4) (see model definition in the methods section). This oscillator can synchronize with periodic external stimuli, a feature that has been exploited by a model capable of beat tracking in complex musical rhythms [8,34]. The oscillator’s periodic activity phase-locks with external periodic stimuli close to its fundamental frequency and also at integer-ratio relationships [33].

**Fig 1:**
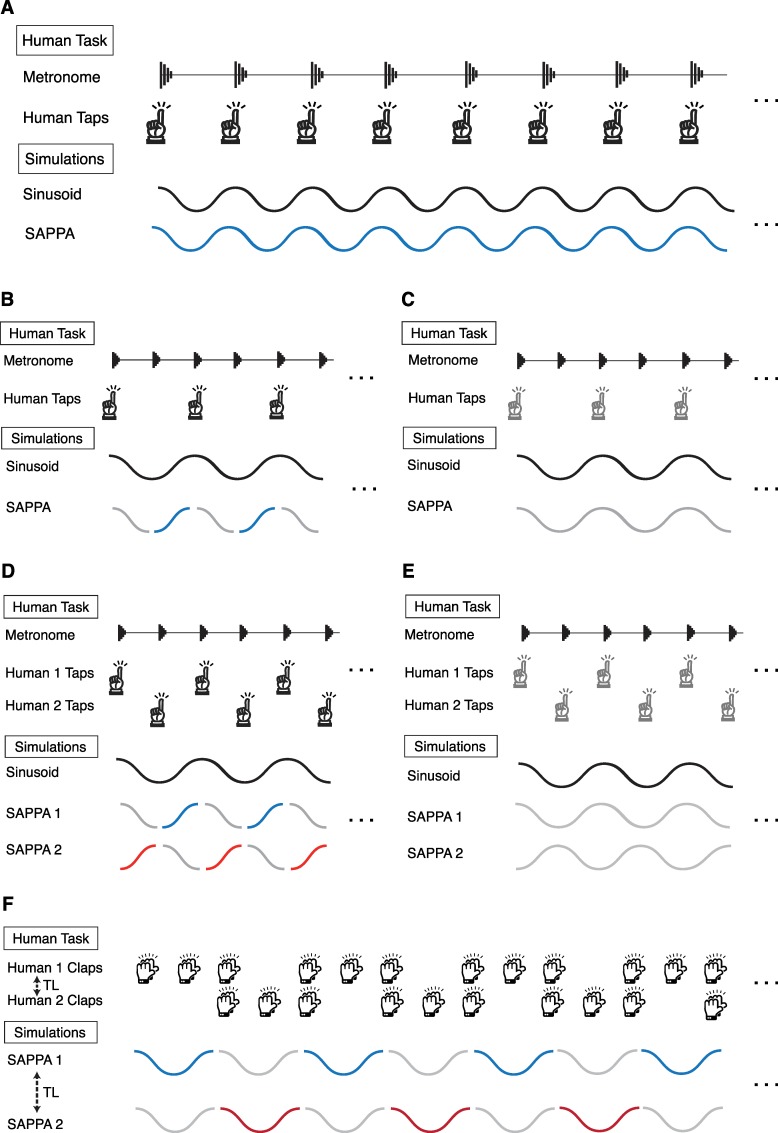
Illustration of the synchronization tasks and corresponding simulation experiments. (A) The task simulated in Experiment 1, in which a person synchronizes with a metronome (top). Illustration of our simulation, in which our model synchronizes with an external sinusoidal stimulus (bottom). (B) The first task simulated in Experiment 2, in which one musician taps to every other metronome beat while listening his or her own taps (top). Illustration of our simulation in which a SAPPA model synchronizes with an external sinusoidal stimulus (bottom). Blue colored part of the model’s activity indicates that the model is receiving its own non-delayed activity as input in addition to the external sinusoid, and gray colored part indicates that the model only receives the external sinusoid as input. (C) The second task simulated in Experiment 2. This task is the same as the first one described in (B), except that the musician did not hear his or her own taps (top). Illustration of our corresponding simulation (bottom). The gray lines indicate that the model only receives the external sinusoid as input. (D) The third task simulated in Experiment 2, in which two musicians alternately tap with a metronome while listening to their own taps and the other musician’s taps (top). Illustration of our simulation where two models synchronize with an external sinusoidal stimulus (bottom). Blue and red colored parts of the model’s activity indicate the time window where the model’s non-delayed activity is used as input for both models in addition to the external sinusoid, while grayed part indicates the time window when the model receives the non-delayed activity of the other model in addition to the external sinusoid as input. (E) The fourth task simulated in Experiment 2. This task is the same as the third one described in (D), except that the musicians did not hear their own or each other’s taps (top). Illustration of our corresponding simulation (bottom). The grayed cycles indicate that the models only receive the external sinusoid as input. (F) The task simulated in Experiment 3, in which two musicians clapped a rhythm alternately (top). Illustration of our simulation where two models oscillate while alternately receiving each other’s activity as input (bottom). Blue and red cycles indicate the model whose activity is received by both models as input, while gray cycles indicate that the model’s activity is not received as input by either model. TL stands for transmission latency.

In contrast to the models previously described by Large and colleagues using a network of coupled oscillators [8,33–34], our model consists of a single oscillator. To simulate periodic synchronization, we add delayed recurrent feedback to this single oscillator. Such delayed recurrent feedback is essential for a model of synchronization, as no neural process is instantaneous [31]. We refer to our model as the Strong Anticipation in Periodic Perception Action (SAPPA) model (see model definition in the methods section). Below we describe three simulation experiments (see Fig 1) based on published data corresponding to three distinct behavioral studies.

In our first experiment, we use the SAPPA model to reproduce the data from a study in which musicians and non-musicians synchronized their tapping with an isochronous metronome across a wide range of tempi, representing the simplest form of synchronization task [4] (Fig 1A). Specifically, we hypothesize that the delayed recurrent feedback in the SAPPA model would result in an increasing anticipation tendency for the longer stimulus periods. Additionally, the smaller anticipatory tendency observed in musicians compared to non-musicians’ anticipatory tendency would be achieved by reducing the amplitude of the delayed recurrent feedback.

In our second experiment, we reproduce the data from a study in which two musicians alternately tapped to a metronome [12]. We are specifically interested in the data from two tasks where musicians tapped at every second metronome beat alone (Fig 1B and 1C) or alternated this tapping with another musician as a duet (Fig 1D and 1E). In both cases, their performance was measured in two conditions, with and without auditory feedback from tapping. The results show a smaller anticipatory tendency, in both solo and duet settings, when musicians heard their own taps compared to when they did not. We hypothesized that the lack or presence of non-delayed feedback in our model can simulate auditory feedback conditions, affecting the size of the anticipation.

In our third experiment, we reproduce behavioral data from duet performance with TLs (varying from 3ms to 78ms for different trials) where two musicians alternately clapped the same rhythmic pattern without a metronome [14] (Fig 1F). Latencies longer than 20ms caused the musician starting the pattern to lag the musician finishing the pattern, while latencies shorter than 10ms caused the musician starting the pattern to clap ahead of the other musician’s last clap. This resulted in the collective tempo gradually slowing down for the longer TLs and speeding up for the shorter ones. We hypothesized that our model’s anticipation would be affected by longer TLs, resulting in lag between two synchronizing SAPPA models and a slowing tempo.

Perception-action coordination tasks capture how external and internal factors affect synchronization. The strong anticipation hypothesis explains synchronization with dynamical systems receiving external stimuli and delayed recurrent feedback. Our interest is to test whether a mathematical model of strong anticipation can be configured for solo and duet settings to perform a variety of synchronization tasks, and if so whether it could explain behavioral patterns observed in human data. This would demonstrate that the strong anticipation hypothesis accounts for complex biological phenomena like perception-action coordination.

## Results

### Experiment 1: Individual tapping in synchronization with an isochronous stimulus

**Fig 2:**
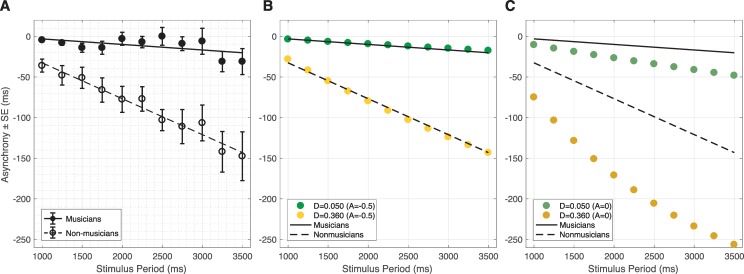
Dynamical systems model of anticipation when musicians and non-musicians synchronize with an isochronous stimulus. (A) The anticipation (mean values with error bars representing the standard error of the mean) in musicians and non-musicians tapping with an isochronous metronome while listening their own taps. The regression lines for the mean values are also shown. (B) The anticipation obtained when the musician (green dots) and non-musician (yellow dots) SAPPA models were stimulated by an external sinusoid while also receiving their own non-delayed activity as input (*A* = -0.5; see model definition in the methods section). (C) The anticipation obtained when the musician (gray-green dots) and non-musician (gray-yellow dots) SAPPA models were only stimulated by an external sinusoid and did not receive their own non-delayed activity as input (*A* = 0; see model definition in the methods section). In all simulations *τ* = 0.222 seconds. The *D* parameter differentiates the musician and non-musician models. The same regression lines for the behavioral data are shown in both (A), (B) and (C) for comparison purposes (see Supplementary S1 Fig for the model’s behavior with a square wave input).

We simulated the model to represent an individual tapping with an isochronous stimulus (see Fig 1A), resulting in an anticipatory tendency. Fig 2A shows the linear regression we performed on the human data described by Repp and Doggett [4]. We stimulated our model with a periodic external sinusoid and measured its anticipation (see model definition and parameter analysis in the methods section) with respect to the external sinusoid. In order to observe the relationship between anticipation and stimulus period length, we optimized our model parameters (see parameter analysis in the methods section) to simulate the anticipation shown by musicians and non-musicians, as a function of IOI [4]. In Experiment 1, a single SAPPA model was stimulated by an external sinusoid while also receiving its own non-delayed activity as input. The range of oscillatory frequencies tested in Experiment 1 ranged from 0.29Hz to 1Hz (see the methods section for a full description of the model parameters and experimental conditions). This contrasted with Experiments 2 and 3, where the same model was stimulated differently, at a smaller set of frequencies.

We ran our simulations for stimuli with periods of 1000, 1250, 1500, 1750, 2000, 2250, 2500, 2750, 3000, 3250, and 3500ms. The SAPPA model always had a recurrent feedback delay with length of 222ms. To simulate the musician and non-musician data, the SAPPA model had *D* = 0.05 and *D* = 0.36, respectively, where the parameter *D* is the amplitude of the delayed recurrent feedback in the SAPPA model (see model definition and parameter analysis in the methods section). Using the same recurrent feedback delay length, we were able to make the slope of the anticipation, as a function of IOI, more negative by increasing the amplitude of *D*. Fig 2B shows how our simulated anticipatory tendencies align with the line of regression on the behavioral data for musicians and non-musicians in the study by Repp and Doggett [4]. Fig 2C uses the SAPPA model to predict how behavioral data would look if musicians and non-musicians carried out the same task only listening to the metronome and not receiving auditory feedback about their taps. Under this condition, the SAPPA model predicts that the asynchrony in musicians and non-musicians would become more negative, compared to when they received auditory feedback about their taps.

In this simulation, the following parameters were used: *A* = -0.5 (non-delayed feedback amplitude), *τ* *=* 0.222 seconds (delay length), and *D* = 0.05 and *D* = 0.36 (delayed feedback amplitude) for the musician and non-musician SAPPA models, respectively. The stimulating sinusoid had an amplitude of 1, while *A =* -0.5. This means that the model was forced more strongly by the external sinusoidal stimulus than by its own activity and that the SAPPA model’s phase-locking behavior was determined by the phase of the external sinusoid. Also, since recurrent feedback terms were negative (*A =* -0.5), the delayed recurrent feedback shifted the phase of *F* in a negative direction with respect to exp(*i*2*π f*_*s*_ *t*). This means that the SAPPA model’s actions were more aligned with $F=\frac{exp(i2\pi \;f_{s}\;t)+Az}{\left|exp(i2\pi \;f_{s}\;t)+Az\right|}$, thus resulting in reduced anticipation compared to when *F* = exp(*i*2*π f*_*s*_ *t*) (see the methods section for a thorough description of the model’s dynamics, parameters, and discussion of how different model components interact with each other).

### Experiment 2: Interpersonal synchronization during alternating paced tapping with or without auditory feedback

Experiment 1 showed that our model can capture the anticipation observed in musicians and non-musicians in a simple metronome tapping task. Next, we examined how the SAPPA model could perform the more complex tasks in the study by Nowicki and colleagues [10]. This task was carried out by solo musicians and also by musician duets. In the solo task, musicians tapped every other beat in synchrony with a metronome while hearing their own taps (feedback-on, Fig 1B) or only hearing the metronome (feedback-off, Fig 1C). In the duet task, pairs of musicians alternately tapped with a metronome while hearing their own and the partner’s taps (feedback-on, Fig 1D) and also while only hearing the metronome (feedback-off, Fig 1E). The same model and parameters used in Experiment 1 were used in Experiment 2, except for the *f* term, which was varied in Experiment 1 but was always 1Hz in Experiment 2, matching the frequency of the stimulating external sinusoid. Another major difference was the way in which the model was stimulated. Similar to Experiment 1, each SAPPA model in Experiment 2 was constantly stimulated by an external sinusoid. However, in Experiment 2 in the solo condition with feedback-on, the model received its own non-delayed activity as input only during half of each cycle. Moreover, in Experiment 2 in the duet condition with feedback-on each model received its own non-delayed activity as input during one half of each cycle and the other model’s non-delayed activity during the other half of the cycle. Finally, in Experiment 2 in the feedback-off condition, the models were only stimulated by the external sinusoid (see the methods section for a full description of the model parameters and experimental conditions).

**Fig 3:**
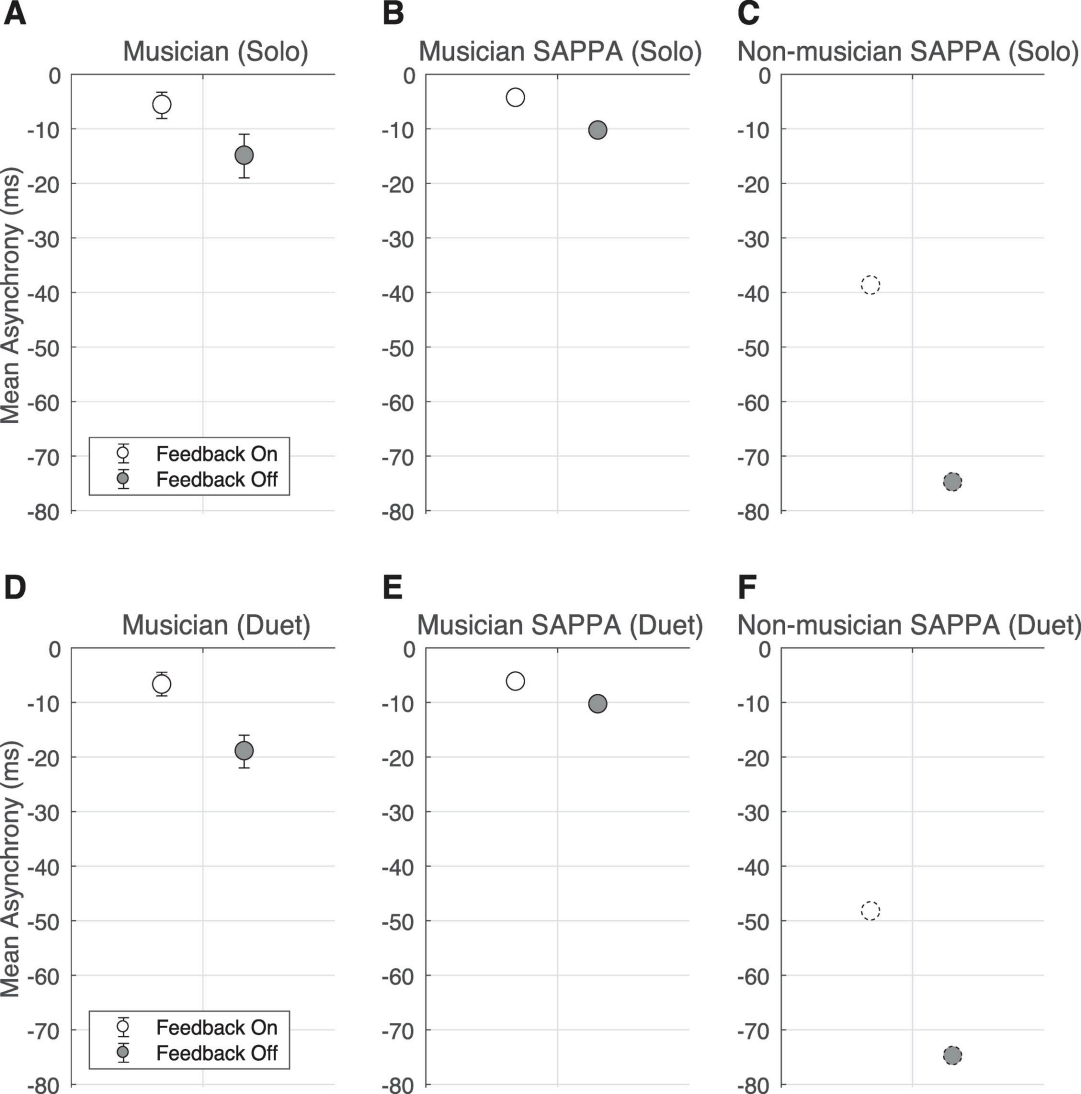
The effect of auditory feedback on anticipation when musicians synchronize with an isochronous metronome alone or with a musician partner. (A) Behavioral measurements when a single musician taps every other beat in synchrony with a metronome for the feedback-on and feedback-off conditions (mean asynchronies with error bars representing the standard error of the mean). (B) The musician SAPPA model’s anticipation when synchronizing with an external sinusoid, while receiving (feedback-on) or not receiving (feedback-off) its non-delayed activity as input every other beat. (C) The non-musician SAPPA model’s anticipation when synchronizing with an external sinusoid, while receiving (feedback-on) or not receiving (feedback-off) its non-delayed activity as input every other beat. Dotted contours around circle data points indicate that this is a prediction that can be tested with behavioral data from non-musicians. (D) Behavioral measurements when two musicians tap every other beat in synchrony with a metronome for feedback-on and feedback-off conditions (mean asynchronies with error bars representing the standard error of the mean). (E) The anticipation when two musician SAPPA models synchronize with an external sinusoid, while alternating (feedback-on) or not receiving at all (feedback-off) each other’s non-delayed activity as input every beat. (F) The anticipation when two non-musician SAPPA models synchronize with an external sinusoid, while alternating (feedback-on) or not receiving at all (feedback-off) each other’s non-delayed activity as input every beat. Dotted borders around data points indicate that this is a prediction that can be tested by collecting behavioral data from non-musicians.

Fig 3A shows that the anticipation exhibited by musicians when solo-tapping on every other beat to an isochronous metronome was larger without auditory feedback from their own taps. The results of our solo-tapping simulations for the musician and non-musician SAPPA models are illustrated in Fig 3B and 3C, respectively (the SAPPA models used in this simulation have the same parameters found in Experiment 1; see the methods section for details about how we carried out this simulation of the solo task). We observed that the SAPPA model’s anticipation was smaller when the model did receive its non-delayed activity as input, compared to when only receiving the external sinusoid as input. While no study has yet investigated non-musicians’ asynchronies in this solo-tapping task, our non-musician SAPPA model predicts that non-musicians’ asynchronies would be larger than musicians’ asynchronies. Our SAPPA model also predicts that, similar to musicians, the non-musicians’ asynchronies will be smaller when individuals can hear their own taps along with the metronome, compared to when they only hear the metronome.

Fig 1D and 1E show the duet behavioral task, which consists of two musicians tapping in alternation by each synchronizing with every other beat of an isochronous metronome. Fig 3D shows that, similar to the solo task results, the anticipation in musicians for the duet task was larger when they could not hear their own or the partner’s actions. The results of our duet-task simulations with the musician and the non-musician SAPPA model are illustrated in Fig 3E and Fig 3F, respectively (the SAPPA models used in this simulation have the same parameters found in Experiment 1; see the methods section for details about how we carried out this simulation of the duet task). We observed that the SAPPA model’s anticipation was smaller when models alternatingly received each other’s non-delayed activity as input, compared to when they only received the external sinusoid as stimulus. No study has yet investigated non-musicians’ asynchronies in this duet-tapping task, but we can use the non-musician SAPPA model to make predictions. Compared to anticipation in musicians, the SAPPA model predicts that anticipation in non-musicians would be larger. The SAPPA model also predicts that, similar to musicians, the non-musician anticipation will be smaller when pairs of individuals can hear each other’s alternating taps along with the metronome, compared to when they only hear the metronome.

### Experiment 3: Interpersonal synchronization during rhythm-clapping alternation in the presence of transmission latencies

In Experiment 2 we coupled pairs of SAPPA models to simulate the flow of auditory information between partners performing a paced alternating tapping task. Here we conduct another simulation for the data described by Chafe and colleagues [14], by further extending our model and task configuration. Their study examined how TLs between duet partners affected synchronization while they alternately clapped a rhythmic pattern (see Fig 1F). The two novel features in their task were that they added TLs to the information exchange between duet partners who performed the task in two different rooms, and that they used a rhythmic pattern for alternating clapping instead of single-beat alternation. The same model and parameters used in Experiments 1 and 2 were used in Experiment 3. The main differences were, again, the way in which the model was stimulated and the oscillatory frequency. In contrast with Experiments 1 and 2, the SAPPA models in Experiment 3 were never stimulated by an external sinusoid. Instead, pairs of oscillators (*f* = 1.5Hz) stimulated each other in an alternating fashion every cycle (see the methods section for a full description of the model parameters and experimental conditions).

**Fig 4:**
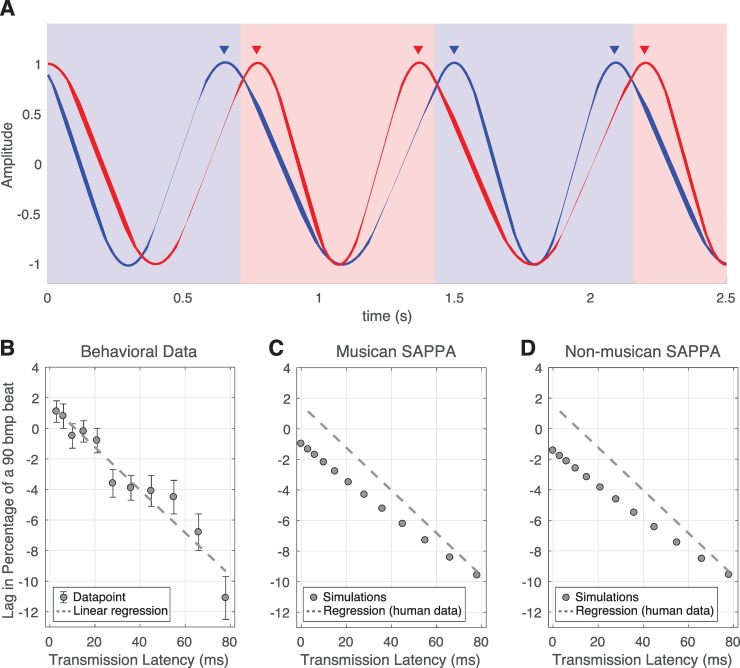
The effect of transmission latencies on the anticipation of pairs of musicians alternatively clapping a rhythm. (A) Illustration of the dynamics observed during our simulations in the presence of a TL between two synchronizing SAPPA models. The alternating blue and red background colors indicate which model’s activity is used as input to both models. The arrows show the end points of cycles for both models. Note how the passive model lags the active model in turn at the end of the cycle, due to the presence of the TL. (B) The lead and lag between musicians (measured as the percentage of a 90 bpm beat) as a function of TLs (mean values with error bars representing the 95% variance), with the linear regression on the behavioral data. (C-D) The lead and lag between pairs of musician (C) and non-musician (D) models, with the linear regression from the behavioral data (B) for comparison purposes.

In the results by Chafe et al. [14], for TLs smaller than 10ms, the musician starting their rhythm showed a tendency to slightly lead the musician finishing a turn. With TLs greater than 10ms, the musician starting their turn lagged the musician finishing a turn. A similar pattern of behavior was observed as a result of TLs between the two oscillators. Fig 4A illustrates our simulations with two oscillators that alternated cycles based on which oscillator served as the ‘active’ one. The colored background indicates the oscillator whose activity was used as the input for both oscillators (see the methods section for more details about how we carried out this simulation). In the example shown in Fig 4A, the oscillator initiating its active cycle tended to lag the endpoint of the other oscillator as a result of the TL. In human data, these asynchrony measures grew as a function of TL, as shown in Fig 4B. Fig 4C and 4D show our simulations with musician and non-musician SAPPA models, respectively, where the lag between coupled models showed a growth similar to the one observed in the behavioral study. The main difference between the behavioral data (Fig 4B) and our simulations (Fig 4C and 4D) is that in our simulations TLs smaller than 10ms did not result in positive values. No existing studies have addressed how non-musicians perform in this task. Our non-musician SAPPA model predicts that, compared to musicians, the effect of TLs on non-musician synchronization behavior will be similar to that observed for musicians, indicating that TLs consistently affect synchronization between two clapping individuals, independent of musical expertise.

## Discussion

### Delayed recurrent feedback results in anticipatory tendencies during paced periodic action

Our model shows that asynchronies become smaller when the length of the recurrent feedback delay is shortened (see parameter analysis in the methods section). This is a result of the fact that *z* and *z*(*t—τ*) become maximally aligned as *τ* shrinks. Consistent with the observations by Ciszak and colleagues [35], our simulations show that the phase difference between *z* and *z*(*t—τ*) is what causes anticipatory behavior. This behavior is similar across different types of external periodic inputs (for example, see Supplementary S1 Fig for the model’s behavior when the external input is a square wave instead of a sinusoid). It is interesting to consider whether the observed phase difference between *z* and *z*(*t—τ*) is similar to the communication delays that exist in the physiology of the sensorimotor system. In principle, today's imaging techniques can support the quantification of actual conduction delays between neurons located in the auditory cortex, the basal nuclei, the cerebellum, premotor and motor cortex, as well as extremities and effectors (e.g., fingers). For example, high resolution neuroimaging methods and the physical models of axonal conduction delays may allow for the estimation of these delays through integration over populations [36]. However, these areas contain vast numbers of neuronal connections which likely differ from each other in their functional pathways. Thus, detailed estimation of these delays at microscopic levels may not easily translate to macroscopic representation of neuronal population behaviors. Given that the recurrent feedback delay length is arbitrarily set in our model, the recurrent feedback delay length described here should be considered a representation of the potential function of neural transmission delays in a collective fashion.

### Experiment 1: Individual tapping in synchronization with an isochronous stimulus

In the SAPPA model Eq (5), a larger *D* increases the amplitude of the delayed recurrent feedback. These different amplitudes have an effect on the anticipatory mechanisms of the model. *D* is divided by the frequency *f* in Hz. As *f* becomes smaller, $\frac{D}{f}$ becomes exponentially larger. Hence, the delayed recurrent feedback is amplified as a function of the stimulus interval, which in turn results in the growing anticipation. Physiologically, this means that the $\frac{D}{f}$ parameter could indicate the amplitude of neural activity that one processes in addition to the external stimulus. This amplitude becomes larger as the stimulus becomes slower.

Our model was able to reproduce the mean anticipatory dynamics of musicians and non-musicians tapping with IOI periods between 1000ms and 3500ms. This was due to the smaller *D* that made the musician SAPPA model anticipation curve have a less negative slope as a function of stimulus period length, compared to the larger *D* that resulted in a more negative slope of non-musician SAPPA model. Since the amplitude of the delayed recurrent feedback in the non-musician SAPPA model is overall larger compared to the musician SAPPA model, $\frac{D}{f}$ makes the delayed recurrent feedback grow more in amplitude as a function of longer stimulus period. As discussed above, directly relating this *D* parameter to the real neural processes might be difficult because it captures the cumulative effect of multiple interacting neural delay processes [37]. What we can say is that the larger *D* value in the non-musician model causes larger amplitudes in the oscillators compared to the musician model.

We assumed that a linear regression on the behavioral data could fairly characterize the anticipatory dynamics observed in musician and non-musician data (Fig 2A). This assumption is a limitation: anticipatory timing does not grow indefinitely as a function of IOI. Experiments where humans synchronize taps with a metronome of IOIs longer than 3500ms show that some taps precede the stimulus while others follow it, reducing the mean asynchrony [3], or even showing mean positive asynchronies for IOIs greater than 5000ms [5]. Hence, the relationship between metronome IOI and human asynchronies are clearly non-linear. Non-linearities are a common feature in systems with delayed recurrent feedback [38]. As a result, our model’s behavior is non-linear, a feature that is more obvious in our simulation of non-musician anticipation than musician anticipation (Fig 2B and 2C). These non-linear features could be used to expand the SAPPA model by making it show positive asynchronies in simple tapping tasks, like people do when stimulated by IOIs greater than 5000ms [5]. Because of these non-linear features, the SAPPA model has potential to explain both positive and negative asynchronies observed when people tap with metronomes of different IOI lengths.

Behavioral studies have consistently shown that anticipation when tapping with a metronome differs between musicians and non-musicians [2,4–5]. Our model offers an explanation as to what underlying mechanisms could give rise to these differences. Here, only the *D* parameter was different between our musician and non-musician models, with *D* being smaller in the musician model. Moreover, the simulation in Fig 2C predicts that the anticipation will be larger for both musicians and non-musicians synchronizing with a metronome without listening their own taps, compared to when they listened their own taps (Fig 2A and simulation in Fig 2B). This prediction made by the SAPPA model could be tested by collecting empirical evidence from musicians and non-musicians. Furthermore, by changing its parameters, our model allows us to make even more predictions, beyond the ones made in Fig 2C.

Compared to the musician model, the larger *D* parameter in the non-musician model could be equivalent to a scenario where non-musicians listen to their internal processes more than musicians. In other words, in the non-musician’s brain the strength of internal feedback is larger compared to the musician’s brain, resulting in processing of an internal signal in addition to processing of external stimuli or behavioral adjustments. In this analysis we focused on the *D* parameter, but the *β* parameter in the SAPPA model would have had a similar effect (see model definition in the methods section for a full description of the model parameters). Electrophysiological findings from investigations on cortical oscillatory amplitude support this observation. Compared to resting conditions, non-musicians listening to music show larger oscillatory amplitudes only in the delta band (between 0.5Hz and 4Hz) [39], which overlaps with the frequency range of stimuli tested in our Experiment 1. In contrast, musicians listening to music show synchrony in a more diverse set of neural oscillation frequencies, including delta and gamma (greater than 30Hz) bands [39]. Our model’s *D* parameter could be inversely proportional to the number of neural oscillation frequency bands observed when listening to music compared to resting conditions. Other neuroscientific research regarding the question of how musical expertise affects synchronization indicates that, compared to non-musicians, musicians show enhanced executive function and activation of the supplementary motor area [40], are better able to imitate hand gestures [41], and show greater connectivity between premotor and striatal brain areas during beat perception [42]. Hence, musical expertise affects synchronization. Further, Riley and colleagues [43] hypothesize that behaviors involving precision, like perception-action coordination, rely on synergy formation, which is associated with a reduction of the degrees of freedom that a person has to handle cognitively during a task. Through training, synergies associated with synchronization may be greater in musicians than in non-musicians. The *D* parameter in the SAPPA model could be understood as related to the number of degrees of freedom that a person has to handle during perception-action coordination, since *D* had a smaller value in the musician SAPPA model compared to the non-musician SAPPA model.

Our model lacks variability in its behavior when synchronizing with an isochronous stimulus. When people tap with an isochronous stimulus, a mean negative asynchrony is observed with a large variability around it [9]. This means that some people may exhibit mean positive asynchronies (e.g. reaction to the stimulus rather than anticipating). The current SAPPA model can only describe the mean anticipatory tendency observed when averaging data across individuals. Variability in the SAPPA model could be achieved with simulations where the *D* parameter is varied using a gaussian distribution centered around the *D* values we found for musicians and non-musicians. Bååth [3] used mixed gaussian distributions to simulate the anticipation variability seen in the behavioral experiments similar to those in Repp and Doggett [4]. Using gaussian distributions to add variability to the SAPPA model would also allow models of individual participants to have different parameters. One could test whether the SAPPA model’s *D* parameter is related to individual’s oscillatory power or years of musical training. The SAPPA model is currently unable to explain anticipatory tendencies or positive asynchronies observed outside the IOI range between 1000ms and 3500ms. Future investigations should test the SAPPA model for anticipation outside the IOI range we investigated in this study. When tapping with IOIs longer than 3500ms, people may show mean positive asynchronies, while mean negative asynchronies are observed when tapping with IOI lengths shorter than 1000ms [5]. We already discussed in the previous paragraphs that the SAPPA model’s non-linearities could explain the positive asynchronies observed for IOIs longer than 3500ms. Although beyond the scope of the current investigations, we did observe that for IOIs shorter than 1000ms, the SAPPA model shows a negative asynchrony, consistent with behavioral data.

### Experiment 2: Interpersonal synchronization during alternating paced tapping with or without auditory feedback

In the solo and duet conditions, our musician SAPPA model showed less anticipation when musicians tap every other metronome beat hearing their own actions compared to when only hearing a metronome. When the SAPPA model synchronizes with its own non-delayed activity in addition to the external sinusoid, the input *F* is the sum $F=\frac{exp(i2\pi \;f_{s}\;t)+Az}{\left|exp(i2\pi \;f_{s}\;t)+Az\right|}$ (as shown by Eq (7) in model definition in the methods section). The smaller anticipation when $F=\frac{exp(i2\pi \;f_{s}\;t)+Az}{\left|exp(i2\pi \;f_{s}\;t)+Az\right|}$ as opposed to when *F* = exp(*i*2*π f*_*s*_ *t*) (i.e. only the external sinusoid) is caused by the instantaneous phase shift that *F* suffers when *z* gets subtracted from the external sinusoid exp(*i*2*π f*_*s*_ *t*), causing *F* and *z* to be more phase-aligned. In contrast, when only synchronizing with an external sinusoid, the delayed recurrent feedback $\frac{D}{f}z(t-\tau )$ (see Eq (5) in model definition in the methods section) linearly adds with *F* = exp(*i*2*π f*_*s*_ *t*), resulting in a delayed phase shift, causing *F* and *z* to be less phase-aligned.

We simulated anticipation using the musician and the non-musician SAPPA models. As shown in Fig 3, the musician SAPPA model was able to show smaller anticipation in the feedback-on compared to the feedback-off condition in both solo and duet tasks. While the task by Nowicki and colleagues [10] has not yet been tested with non-musicians, our simulations predict that the non-musician anticipation will generally be larger than the musician anticipation observed across tasks and conditions. We speculate that this prediction is highly likely, because it has been previously reported that non-musicians’ asynchronies are larger than musicians’ asynchronies [4]. By collecting data from non-musicians carrying out this task one could test the validity of our model’s predicted behavior.

Currently, the SAPPA model does not include modules that represent variabilities over time across individuals in a sample population. This means that it is limited in its ability to explain long-range temporal dynamics. Hence, anticipatory dynamics are limited to a local time scale. Incorporating noise in our model is beyond the scope of the work we present here, because the nature of noise as well as its sources and mechanisms remain largely unknown. As previously discussed, Bååth [3] has modeled the noise associated with anticipation in the simplest metronome tapping task using a mixed gaussian distribution. To our knowledge, no studies have demonstrated how neural mechanisms and their intrinsic noise might be related to synchronization phenomena and anticipation. Variations in the environment and task requirements are also likely to add different types of noise that influence the overall system behavior. To effectively simulate different noise sources, it would be necessary to first study the distributions and mechanisms underlying variability in human adaptive behavior, using a large corpus of empirical data. Additionally, when quantifying how noise affects synchronization at local and global time scales, researchers should carefully consider that asynchronies tend to fluctuate on long-range time scales, which can affect the interpretation of behavioral data analysis and computational models [44].

Because the two musicians in the duet task tapped every other beat in alternation, the resulting interpersonal synchronization was anti-phase. Recent studies have used the HKB model [45] (further discussed in the general discussion subsection below) to explain anti-phase interpersonal synchronization [46–48]. The SAPPA model and the HKB models are both able to explain anti-phase synchronization and anticipation at different phase relationships. Future investigations could analyze similarities and differences between these models when they synchronize with external periodic stimuli.

### Experiment 3: Interpersonal synchronization during rhythm-clapping alternation in the presence of transmission latencies

In the simulations in Experiment 3, two types of delay affected synchronization dynamics between coupled dynamical systems. The first one existed in the SAPPA model, as self-referential recurrent feedback delay in Eq (5), which resulted in anticipation (see model definition and parameter analysis in the methods section). The second one, TL, was introduced as the external informational delay in the communication between two oscillators, which is extrinsic to Eq (5). Our simulations integrate these two separate delays to explain how internal and external feedback in our model affect synchronization. Experiment 1 results showed that the first delay resulted in the anticipation, while the second delay can cancel the anticipatory behavior, resulting in disrupted synchronization. Our simulation results show that TLs similarly affected the synchronization of the musician and non-musician SAPPA models (Fig 4C and 4D). This suggests that TLs affect synchronization independent of musical expertise. Future experiments can test our model’s prediction of non-musician behavior by collecting data when non-musicians carry out the task introduced by Chafe and colleagues [14].

In Experiment 3, pairs of oscillators (*f* = 1.5Hz) stimulated each other in an alternating fashion every cycle. There was no external sinusoid stimulating the oscillators in this experiment (see setup, procedures and measurements in the methods section for a complete description). One caveat to our current model, is the fact that it cannot explain the positive values observed in existing human behavioral data (Fig 4C and 4D). In the experiment by Chafe and colleagues [14], pairs of participants synchronized in the absence of the natural TL over air (due to wearing headphones), and in the presence of artificial TLs of fixed length. In the real world, synchronizing individuals have to always cope, at least, with the TL associated with sound travelling in air. We speculate that humans cope with such TLs by overestimating the frequency of their actions (i.e. a faster frequency), to make their actions reach the other participant on time. In the experiment by Chafe and colleagues [14], when neither air-travelling sound TL nor artificial TL existed, participants actions anticipated each other. The anticipation, indicated by positive values in the behavioral data in Fig 4B, is likely caused by musicians’ expectation of the TL implicit in the transmission of sound in air, which is effectively removed when the TL is very small (<10ms). Our model is naïve to the TL of sound travelling in air, and thus it was not affected in the same way by TLs smaller than 10ms. In the SAPPA model, the relationship between TL and the natural frequency of the oscillator can only be achieved by adding a bias that makes the SAPPA model complete its cycles before the driving stimulus (another SAPPA model in Experiment 3) completes a cycle. The *f* term, which is a constant dictating the natural frequency of the SAPPA model’s oscillatory behavior in units of hertz (see Eq (5) in the model definition in the methods section), can be altered to explain the positive values in Fig 4B:

$$\hat{f}=f+\Delta$$ (1)

**Fig 5:**
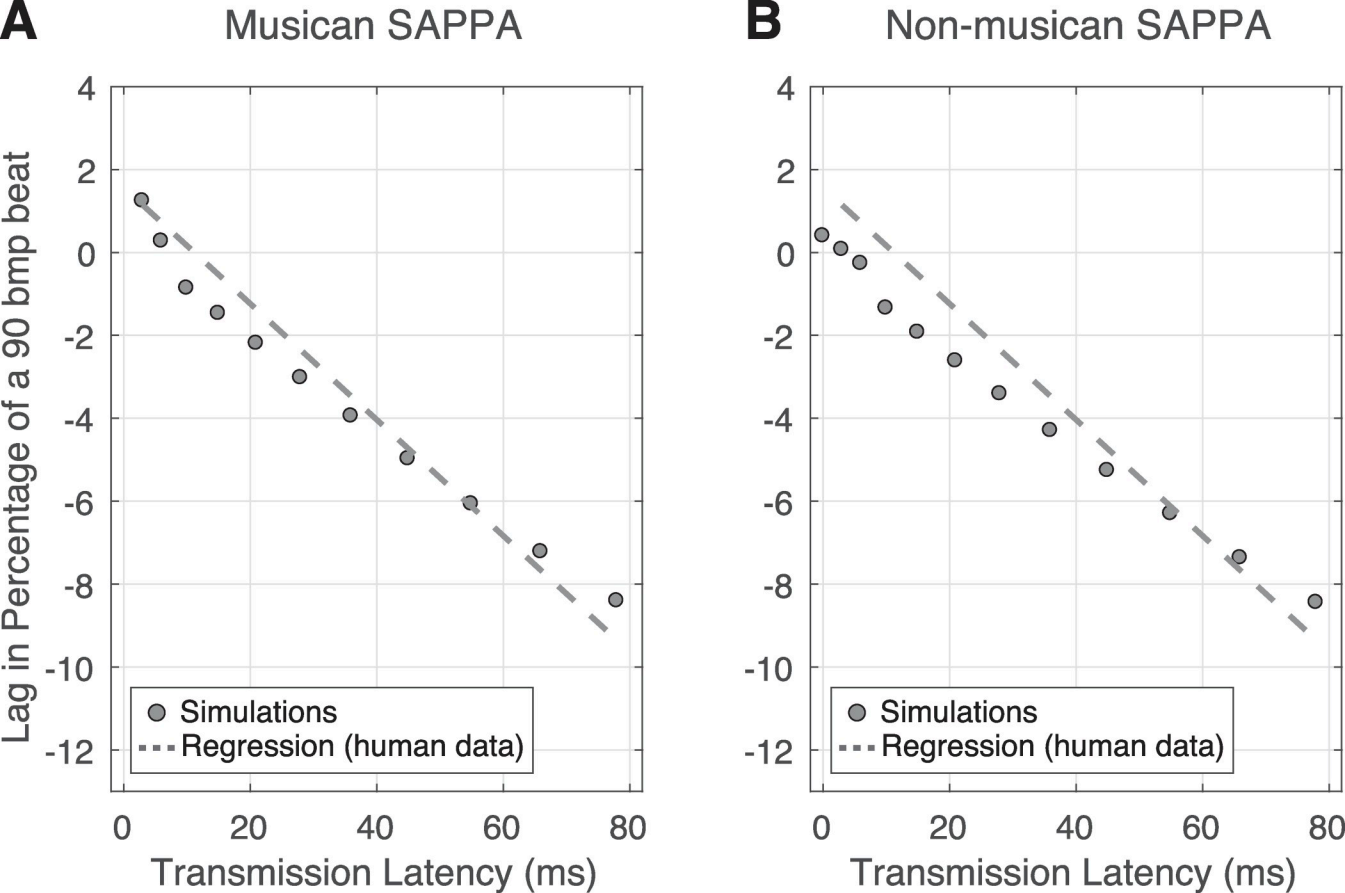
The effect of modified *f* (Eq (1)) on our model’s simulation in Experiment 3 where pairs of musicians alternatively clapping a rhythm in the presence of transmission latencies. The lead and lag between pairs of musician (A) and non-musician (B) models, measured as the percentage of a 90 bpm beat as a function of TLs, with *f* modified as described in Eq (1). The linear regression on the behavioral data from Fig 4B is shown for comparison purposes.

Here, in Eq (1), a positive offset Δ (positive real value bias) can be added to compensate for the effect of the transmission of sound through air. This Δ term would make the SAPPA model’s frequency term slightly faster compared to the external stimulus, resulting in the positive values observed in Fig 4B for TLs smaller than 10ms. Fig 5 shows our model’s resulting behavior when *f* is modified as described in Eq (1).

The behavior in Fig 5 is the result of the frequency detuning introduced in Eq (1). Previous studies show that frequency detuning and delayed coupling between synchronizing HKB systems (see the General discussion below for more information about the HKB model) result in anti-phase solutions that are less stable than in-phase activity [32,49]. The behavioral data by Chafe and colleagues [14] supports these observations, since musicians performed the same clapping rhythm in-phase, in an alternating fashion. As the transmission delay grew, in-phase synchronization became harder for pairs of synchronizing musicians. To cope with different TL lengths, musicians had to re-adjust the frequency of their actions every cycle to continue in-phase, but very long TLs made in-phase synchronization impossible.

Humans may overestimate the frequency of their actions when synchronizing with each other to cope with sound air travel TLs. The SAPPA model does not overestimate its frequency, and in the lack of the TL associated with sound air travel, one must manually detune the SAPPA model’s *f* term to result in overestimation. Once this is done the two coupled SAPPA models successfully express the effects of external and internal delays and their interactions in a succinct and integrative manner. Our model can be useful to predict stability of in-phase and anti-phase behavior. Moreover, the SAPPA model could predict how synchronization phenomena like anticipation could be affected by technologies involving TLs, like the ones used for internet-based music performance.

### General discussion

The SAPPA model reproduced anticipatory timing during synchronization at different frequencies, for different rhythms, and in tasks of differing complexity. In Experiment 1 we simulated anticipation for isochronous rhythms at a wide range of frequencies, showing the larger negative asynchrony observed in non-musicians compared to musicians (Fig 2A and 2B). The only difference between the musician and the non-musician model was the *D* parameter, which controls the amplitude of the delayed recurrent feedback and was larger in the non-musician model. In terms of neural underpinnings, the *D* parameter could be inversely proportional to the number of different neural oscillation frequency bands observed when musicians and non-musicians listen to music, compared to resting conditions [39]. Additionally, in Experiment 1 we used our model to predict how the asynchrony would change in an experimental condition where musicians and non-musicians listen and tap with a metronome without hearing their own taps. The model predicts that the asynchrony would grow for both non-musicians and musicians, but the non-musicians’ asynchrony would be larger (Fig 2C). Using the musician model and parameters used in Experiment 1, in Experiment 2 we simulated musicians’ solo and duet synchronization with a metronome in feedback-on and feedback-off conditions. We reproduced behavioral data showing that musicians’ asynchronies are larger in the feedback-off condition compared to the feedback-on condition in both solo and duet synchronization settings (Fig 3A, 3B, 3D and 3E). The smaller asynchrony is caused by a phase shift in the stimulus *F* when *z* gets subtracted from the external sinusoid exp(*i*2*π f*_*s*_ *t*) (see model definition in the methods section). Furthermore, using the non-musician model from Experiment 1 we predicted how non-musicians would perform in this task, projecting overall larger asynchronies compared to musicians, as well as larger asynchronies in the feedback-off condition compared to the feedback-on condition within the non-musician group (Fig 3C and 3F). Finally, using the same model and parameters from Experiments 1 and 2, we demonstrated that the model synchronizes like pairs of musicians do in the presence of a fixed TL and the absence of a metronome stimulus (Figs 4B, 4C and 5A). We explained that the total absence of TL causes humans to speed up their actions, allowing events to be properly timed when sound air travel is considered. Also, using the non-musician model we predicted how pairs of non-musicians would be affected by a fixed TL when synchronizing with each other (Figs 4D and 5B). Our experiments use a musician and non-musician model to successfully reproduce anticipatory timing for different rhythmic stimuli and different tasks, while also making predictions about what not-yet-existing behavioral data in musicians and non-musicians would look like. These simulations reproduce timing characteristics previously observed in behavioral studies, and demonstrate that complex tasks can be performed by the same model by only modifying the stimuli and their frequency.

In the absence of time delays, oscillatory synchronization (e.g., Eq 4) offers only one limited set of explanations for anticipation: the frequency of the oscillation is faster than the frequency of the stimulus [50], underestimation of stimulus period [19], and frequency detuning [51]; *f* > *f*_*s*_. As we see it from a dynamical systems point of view, there are two problems with this explanation. The first is that it is an explanation that requires an explanation: What causes the underestimation of tempo? Secondly, time delays are ubiquitous in the brain and nervous system, thus “adding” time delays to a neural model is not really an adding anything, it is only being more realistic. Neural conduction delays have been conclusively established and measured, and recurrent feedback is equally well-established. Finally, delayed recurrent feedback causes an oscillator with frequency *f* to oscillate at a frequency faster than *f*. Delayed recurrent feedback causes frequency detuning, thus we could say it causes a perceptual underestimation of IOI. Moreover, oscillatory synchronization has intrinsic frequency detuning features (see Large [52] for a theoretical overview of frequency detuning between oscillators).

A large body of literature has studied the dynamics of coordination in a variety of contexts (see [9,23] for reviews). Currently, there are two main views about the neural mechanisms that underlie anticipatory timing. Some researchers have proposed that anticipation results from combining somatosensory and auditory modalities because axonal distances between the hand, the ear, and the brain differ [15–16]. Axonal delays are fixed and the combination of somatosensory and auditory input is likely carried out by assimilation areas of the brain in a bottom-up fashion. While the combination of different sensory modalities can explain the negative phase relationship between human taps and a metronome in principle [17–18], this theory fails to explain many of the key findings, including the fact that anticipation increases with longer metronome periods in the IOI range between 1000ms and 3500ms (see the review of the tapping literature by Repp [9]). The length of time that it takes for the brain to incorporate multiple sensory modalities is likely fixed, so it can only affect the phase relationship between action and stimulus by a constant value, predicting a constant anticipation across different tempi, which is not consistent with behavioral data.

Other researchers explain that anticipation, in general, is the result of neural computations carried out in progressive stages that result in representations [53]. These representations can be about past, present, and future states of the external world. Hence, this view would explain that anticipatory timing emerges from a system’s need to fulfill its representations about the likely future state of the external world [54]. What makes these two views similar is the fact that they see anticipation as the result of a series of staged neural computations that give rise to a representation that is either misaligned with the external stimulus or fulfilled prematurely.

In contrast to these two theories, our model, inspired by the strong anticipation hypothesis, offers a different explanation based on a dynamical systems approach where the properties of the model and its interaction with the external stimulus and surrounding active systems are mathematically described as constrained by universal physical laws [27]. Stephen and Dixon [55] have described that strong anticipation can happen at local and global temporal scales, where local strong anticipation occurs between systems continuously coupled and global strong anticipation is more complex, involving multi-scale interactions (see [56–57] for a thorough discussion of global vs local strong anticipation). The SAPPA model is a clear example of local strong anticipation. All of these observations make the SAPPA model a parsimonious and simple one, whose behavior is the result of interactions with external stimuli, instead of internal representations.

It is noteworthy that other researchers have already built models that could explain and predict human rhythmic coordination behavior by using general physical principles. An important one is the HKB model, which uses a simple equation to explain the possible phase relationships between two coupled oscillatory systems in many contexts, including human intra- and inter-personal synchronization [32]. Because of its focus on relative phase [58], the HKB model is flexible, parsimonious, and a powerful tool to explain and predict periodic human motor behavior [59]. By changing two parameters in a single equation, the HKB model is able to explain transitions between anti-phase (180°) and in-phase (0°) coordination when one person taps both index fingers together [59] or two people swing their feet in tandem [59] at different frequencies. Its full potential has not yet been exploited, since recent bifurcation analyses revealed that the HKB model comprises previously unreported dynamical regimes that could explain an even wider range of human synchronizing behaviors beyond in-phase or anti-phase regimes [49], including squash [60] or butterfly stroke swimming [61]. Although the HKB model has been widely used to explain synchronization behavior, it lacks an explanation of the neurophysiological and biophysical principles that drive synchronization [62]. Some exceptions exist though, since studies have attempted to make the HKB model a more biologically plausible one by explaining how neural connectivity delays affect its behavior [31–32]. While these results are still focused on in-phase and anti-phase phenomena, the added delay allows the HKB model to make predictions about split-brain patients lacking certain modalities of delayed neural cross talk [31] or factors that favor in-phase vs anti-phase stability [32].

Recently a few more models have been proposed that specifically attempt to account for anticipatory synchronization. The first one is the ADaptation and Anticipation Model (ADAM) [63]. ADAM uses phase and period correction to carry out adaptive and anticipatory synchronization with a periodic stimulus that may contain tempo changes [63]. The second, more recent model is proposed by Bose, Byrne and Rinzel [64] proposed as a neuromechanistic model of musical rhythm that, similar to ADAM, corrects its phase and period to find the beat in a periodic stimulus. This neuromechanistic model counts 40-Hz gamma-band oscillatory cycles to quantify how well the model’s beat generator aligns with an external stimulus. Beat synchronization is achieved due to the beat generator’s plasticity, which is informed by the gamma-rhythm count. Both ADAM and the more recent neuromechanistic model compute information from an external stimulus using error correction and extrapolation, and therefore are examples of weak anticipation models. Two other earlier models proposed mechanisms that explained synchronization behaviors. One of them was described by Caceres [65], which used an oscillator described by Large and Kolen [66] for rhythm tracking and generation. Caceres compared his model’s performance with a memoryless model by Gurevich and colleagues [67], providing evidence that oscillators are better at anticipating musical rhythms than memoryless methods [65]. Mates and colleagues proposed that sensorimotor synchronization is influenced by the maximal capacity of temporal integration, which was estimated to be around 3 seconds [2]. This explains why stimuli with IOIs of length greater than maximal capacity of temporal integration cause greater incidence of reactive responses (i.e. after the stimulus onset) and less anticipation compared to stimuli with shorter IOIs.

The SAPPA model is a strong anticipation model because it computes its current state from its physical properties, not through inference [24,27]. Together with the strong anticipation hypothesis and oscillator modeling, our model’s architecture and internal feedback delays could shed light on physical principles of neural mechanisms that underlie anticipation in human behavior. However, it would be useful to further explore these existing models possibly combined with our approach, for scenarios when actions and intentions require substantial temporal deviations from simple integer-ratio based temporal organizations.

Our model is inspired by previous neuroscientific studies and theories explaining how periodic signals like music are processed in the brain. The canonical model of oscillation incorporated in our work has previously been used to explain how people perceive the beat in complicated rhythms [8] and how the brain entrains to simple and complex auditory rhythms signals [8,68]. Hence, the SAPPA model’s Hopf oscillator has been previously used to theorize about brain mechanisms, specifically concerned with the auditory system, rhythm perception, and neural oscillations [8,68]. In our model’s architecture, there are specific components inspired by neuroanatomy and functional units in the human brain involved in synchronization behavior and anticipation. The SAPPA model encodes stimuli in a way that is analogous to what occurs in the auditory cortex in conjunction with timing processing carried out in the basal nuclei and the cerebellum. Additionally, the SAPPA model simulates the looped communication between basal nuclei, sensory areas, and the motor cortex and peripheral systems, all of which are fundamental for synchronization to occur [15,16]. It is important to note that our model works at the level of the oscillatory neural populations, not at the level of single neuron activity. Evidence shows that single neuron dynamics give rise to oscillatory activity at the level of neural populations [69]. Regarding the timing processing, neuromagnetic data by Fujioka and colleagues [70] have shown that listening to an isochronous beat causes periodic amplitude modulation of beta-band (around 20Hz) oscillatory brain activity in the motor and auditory cortices. Furthermore, when beats are accentuated into stereotypical metric patterns, like *waltz* rhythms, the beta-band predicts the beginning of groupings [71]. Findings show that beta-band oscillations are not only associated with sensorimotor functions [72–73] but also the anticipation of event timing [74–75]. Using oscillators to explain neural activity is a low-dimensional approximation of the cortical dynamics observed during synchronization. However, as discussed at the beginning, these high-level representations may be far from microscopic information transactions between neuronal populations.

The model we presented in this paper is general, flexible and parsimonious. As long as the behavior and external factors follow oscillatory patterns, the SAPPA model could simulate the outcome. It uses a canonical model of oscillatory dynamics and shows anticipatory behavior following the general principles of the strong anticipation theory. This makes our model a tool to simplify our understanding of how delayed communication within the human sensorimotor system results in synchronization.

Notably, we used the same model across three different experiments that simulated three different behavioral studies published by different groups at different times. These three behavioral studies employed a variety of tasks and measures, yet, our model was able to capture the behavioral patterns and effects observed across all studies. This demonstrates the versatility and generalizability of our model’s architecture. Our experiments focused on simulating tasks in the context of music because we wanted to test our model in ecologically plausible conditions. The results we presented show that our model can explain human musical behavior, which makes it a tool for the prediction of ecologically-valid anticipation in experiments beyond the ones we discussed here. Our work supports the strong anticipation theory, making new predictions about human behaviors that have not been tested yet. Moreover, our model could aid technologies that assist synchronized action-making in teletherapy with TLs. A better theoretical understanding about how TLs and anticipation interact could lead to a system that dynamically calibrates TLs to help synchronization between humans interacting over the internet. Additionally, knowing how an individual’s neural delays affect synchronization could serve as a biomarker for rehabilitation in personalized medicine [76–77]. Finally, our model could be used to improve telecommunications for synchronized action and have implications in other fields like network music performance and robotics.

## Methods

### Model definition

#### Theoretical background

Negative phase relationships between two systems, such as anticipation, have been observed in the behaviors of coupled dynamical systems like external-cavity diode lasers [78] and FitzHugh–Nagumo systems [79]. The following equations by Voss [80–81] and Ciszak et al. [35] express the general framework of an anticipatory dynamical system with delayed feedback:

$$\dot{x}=h(x)$$ (2)

$$\dot{y}=g(y)+(x-y_{\tau })$$ (3)

Eq (2) describes the stimulus *x*, which has dynamics defined by the function *h*. Eq (3) describes a system *y* with dynamics defined by the function *g*. In Eq (3), the subscript *τ* indicates delayed behavior of the system *y*. This *y*_*τ*_ term is referred to as delay-coupling [81–82]. A negative phase relationship between *y* and *x* is observed when *y* receives *x* and its own delayed activity *y*_*τ*_ as inputs, as described in Eq (3). Importantly, if *y*_*τ*_ is removed from these equations, the anticipatory behavior of *y* in relationship to *x* is no longer observed [82].

Because the SAPPA model simulates periodic synchronization, we used a canonical Hopf oscillator model that allows for synchronization with periodic external stimuli [33]. In contrast to the original model described by Large and colleagues [33], the SAPPA model does not consist of a network of oscillators, but a single oscillator shown in Eq (4).

$$\frac{1}{f}\dot{z}=z\left(\alpha +i2\pi +\beta_{1}{\left|z\right|}^{2}+\frac{\epsilon \beta_{2}{\left|z\right|}^{4}}{1-\epsilon {\left|z\right|}^{2}}\right)+F$$ (4)

In Eq (4), *z* is the state of the oscillator, and *α*, *β*_1_ and *β*_2_ are parameters that control the dynamics of the oscillator while *f* determines the frequency of oscillation. *ϵ* is a parameter that controls the degree of higher-order nonlinear activity in the oscillator and *F* is a stimulus. These dynamics allow a single oscillator to show stable oscillatory activity, even after it is no longer being stimulated, hence showing “memory” [50]. Finally, similar to other strong anticipation models, this model can explain behaviors observed in non-biological systems like Wilson-Cowan networks [33], musical pitch recognition [83], and beat tracking [8].

#### The SAPPA model

Eq (5) shows our model, which is the Hopf oscillator in Eq (4) with *β*_1_ = *β*, *β*_2_ = 0, *ϵ* = 0, and an additional delayed feedback term.

$$\frac{1}{f}\dot{z}=z(\alpha +i2\pi +\beta {\left|z\right|}^{2})+F-\frac{D}{f}z(t-\tau )$$ (5)

Eq (5) in all our simulations has *α* = 1 and *β* = -1 to achieve limit cycle behavior with periodic, unit-magnitude stable and perpetual activity when *F* = 0 and *D* = 0. The oscillator has a fixed frequency determined by the parameter *f* (in Hz). The computations by this oscillator are neuroscientifically inspired. It receives an input *F*, thus encoding and perceiving an external stimulus [8]. It also receives its own delayed activity with amplitude *D* and a delay of *τ* seconds, simulating the delayed communication in the nervous system between basal-ganglia, cerebellum, premotor cortices, motor cortices, and peripheral muscles at extremities and effectors (e.g., fingers), which are inherent in the perception-action cycles [15–16]. We expect the delayed *z* feedback to result in a negative phase relationship between *z* and an external stimulus. In the SAPPA model, summarized by Eq (5), we vary the amplitude of the *D* parameter, while *α* and *β* always have values of 1 and -1, respectively. The value of the variable *f* is selected to match the frequency *f*_*s*_ of a periodic external stimulus (*f* = *f*_*s*_). Because *z* is always unit amplitude, the effect of the delayed recurrent feedback is reduced as *D* shrinks.

The input *F* is a sinusoid with a constant frequency *f*_*s*_. Further, if the SAPPA model is to consider its own behavioral outcome as input (e.g., hearing and feeling one’s own tapping feedback), the *z* activity may be added to the input. Hence, the input *F* may be:

$$F=exp(i2\pi \;f_{s}\;t)$$ (6)

$$F=\frac{exp(i2\pi \;f_{s}\;t)+Az}{\left|exp(i2\pi \;f_{s}\;t)+Az\right|}$$ (7)

F=Az (8)

In Eqs (6) and (7) *f*_*s*_ is the constant frequency of the external sinusoid. Unless otherwise noted, *f*_*s*_ and *f* are the same value (*f*_*s*_ *= f*). Eq (7) normalizes the linear combination of the external sinusoid exp(*i*2*π f*_*s*_ *t*) and *Az*, consistent with behavioral observations of stimulus encoding in synchronization tasks, where changing the amplitude of the stimulus does not affect synchronization [21].

### Parameter analysis

Eq (5) describes *z*’s derivative over time as the linear combination of three terms: the oscillator’s current state *z*, a stimulus *F* (see Eqs (6), (7), and (8) for the different possibilities), and the delayed recurrent feedback *z*(*t* - *τ*). Previous studies have investigated how *z* synchronizes with a periodic stimulus [43, 45]. For this reason, our parameter analysis focuses on how the time length τ and amplitude $\frac{D}{f}$ of the delayed recurrent feedback *z*(*t* - *τ*) affect the anticipation of the SAPPA model.

**Fig 6:**
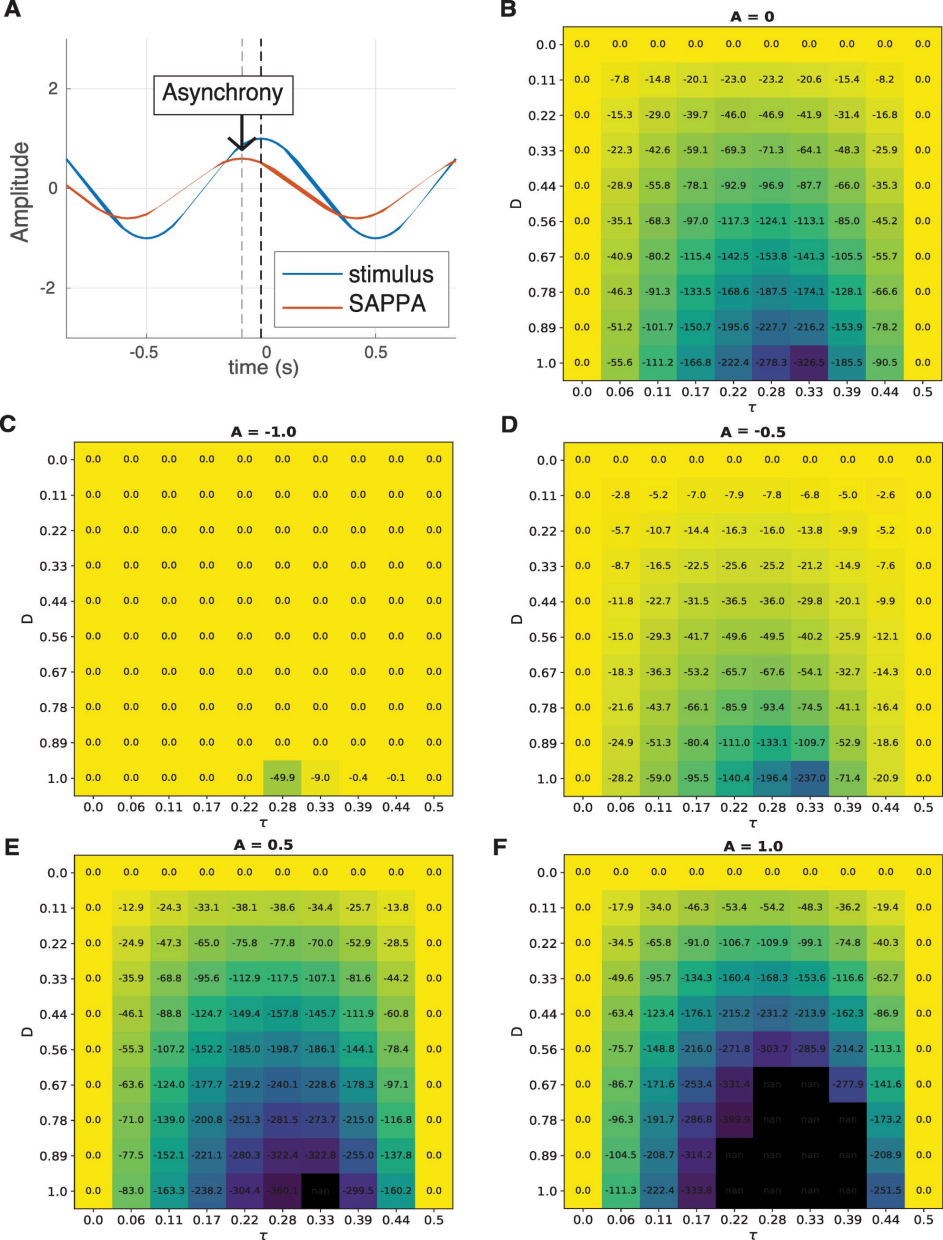
Analysis of the effect that different parameters in the SAPPA model have on its anticipation tendency. (A) Illustration of what the asynchrony between the SAPPA model and the external sinusoidal stimulus can look like, and how it’s measured. (B-F) Analysis of the anticipation as a function of *D* and *τ* in Eq (5), and *A* in Eq (7): (B) *A =* 0, (C) *A =* -1.0, (D) *A =* -0.5, (E) *A =* 0.5, (F) *A =* 1. In these analyses (B-F) the parameter *f =* 1. The numbers in each cell indicate the anticipation (in ms) observed when the SAPPA model synchronized with the external sinusoidal stimulus. A black cell indicates that the SAPPA model did not synchronize with the external sinusoidal stimulus and hence the asynchronies could not be computed. In the analyses (B-F), the asynchrony quickly moves away from zero as 0 <*τ* <0.5, especially when *D* = 1. Additionally, we explored how different initial conditions affect the model’s asynchrony and discontinuities, described in the Supplementary S2 Fig which contains the bifurcation diagram for the SAPPA model when *D* = 1.

Throughout this paper, we fixed the parameters *α* = 1 and *β* = -1 so that *z* shows unit-magnitude behavior when *F =* 0 and *D =* 0. When only *D* = 0, *z* and the external stimulus *F* have the same frequency and phase, so anticipation is absent (see Fig 6), independent of which version of *F* is used (remember Eqs (6), (7) or (8) are the three possible version of *F*). The other parameter in Eq (5) that can be varied is *f*, but since it is only a scale factor that affects the cycling rate of *z*, its effects will be studied later in Experiment 1. In cases when *F* = exp(*i*2*π f*_*s*_ *t*)−*Az*, the magnitude of *A* is another term that can be varied. Hence, the only other terms in Eq (4) that can be varied are *D*, and *τ*.

For this parameter analysis we fixed *f* = 1 to ignore its scaling effect. We analyzed the effect of *D* in the range of values between 0 and 1, which is the dynamic range of *z* when *F =* 0 and *D =* 0, and also the dynamic range of *F*, considering all three Eqs (6), (7) or (8). For *τ*, we analyzed its effect in the range of values from 0 seconds to 0.5 seconds, because the period associated with *f* = 1 is one second and a processing delay with duration closer to the stimulus period length would not make sense during synchronization. To observe the effect of *A*, we also tested *A =* -1, -0.5, 0, 0.5, and 1, which are all values within the dynamic range of *z* (when *F =* 0 and *D =* 0) and *F* (note that when *A =* 0, Eqs (6) and (7) are the same).

We evaluated the anticipation for all possible combinations of *D*, *τ*, and *A* parameters. At the beginning of each simulation, the phase of *z* was initialized to zero. During the first few sinusoidal cycles, the SAPPA model phase-locked with the external sinusoid in order to achieve a state of stable synchronization. From a dynamical systems perspective, ‘synchronization’ refers to the relationship between one system’s actions as closely adhering to another system’s actions [84]. Stable synchronization was observed when the phase difference between the stimulus sinusoid and the model’s oscillators reached a constant value over time. This stable mode is known in dynamical systems theory as a steady state. Fig 6D shows how we calculated the asynchrony between our model’s oscillator *z* and the stimulus sinusoid. After a simulation reached a steady state, we first found the timepoints of the peaks for the real part of the *z* oscillator and the stimulus sinusoid. Then, we subtracted each stimulus sinusoid’s peak time from the nearest *z* oscillator’s peak time. The average peak time difference between the stimulus sinusoid and the *z* oscillator over time was considered the model’s mean asynchrony. Our results are shown in Fig 6. To visualize the results of these simulations, we plotted these results in matrix form, with the x axis indicating the value of *τ*, the y axis the value of *D*, and the color in the matrix cells indicating the asynchrony. We obtained five matrices, one for each of the five values of *A* tested.

### Experiment 1: Individual tapping in synchronization with an isochronous stimulus

#### Behavioral data for simulation

In the task by Repp and Doggett [4], musicians and non-musicians tapped in synchrony with an isochronous metronome in a frequency range between 1Hz and 0.29Hz (corresponding to period durations between 1000ms and 3500ms). They found that the anticipatory tendency increased as a function of metronome period length. Fig 2B shows our models’ anticipation, as well as the linear regression on the behavioral data (Fig 1A in Repp and Doggett [4]). We computed the linear regression lines as the best fit on the anticipation values for musicians and non-musicians.

#### Setup, procedures and measurements

**Fig 7:**
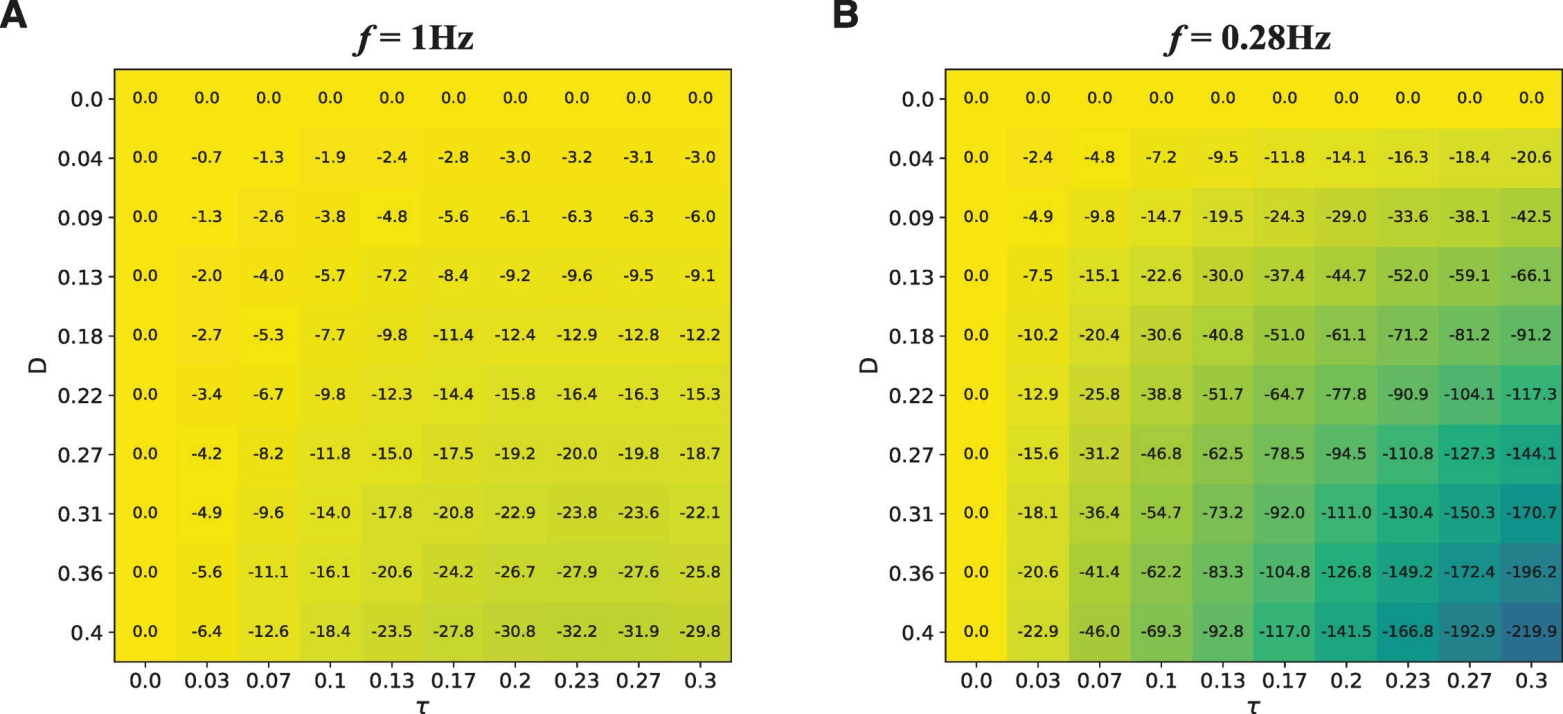
Analysis of the effect that different frequencies in the SAPPA model have on its anticipation. (A) Analysis of the anticipation as a function of *D* and *τ* in Eq (5) when *A =* -0.5 and *f* = 1. (B) Analysis of the anticipation as a function of *D* and *τ* in Eq (5) when *A =* -0.5 and *f* = 0.2857. The numbers in the cells indicate the anticipation (in ms) observed when the SAPPA model synchronized with the external sinusoidal stimulus.

Fig 1A shows the human task and a graphical explanation of our simulation setup that contain the SAPPA model and the input stimulus. In these simulations, the input *F* was *F =* exp(*i*2*π* *f*_*s*_ *t*) *+ Az* because our model “listened” to itself (i.e. received its own instantaneous activity as input). In the parameter analysis section above, we studied the model’s behavior for a frequency of 1Hz. In the human task simulated in this experiment, humans tapped with metronomes of period lengths between 3500ms (approx. 0.2857Hz) and 1000ms (1Hz). Hence, we repeated the parameter analysis with *f* = 0.285 (Fig 7) to observe the model’s behavior at the other end of the spectrum of stimulus frequencies corresponding to this human task. Remember that *f = f*_*s*_. always, unless otherwise noted.

#### Model optimization

To identify the model matching the musician and the non-musician anticipation curves, we identified the set of parameters *D*, *τ*, and *A* that resulted in the best fit between the simulated model’s anticipation and the slopes of the linear regression for the behavioral data (Fig 2B). We found that *A* = -0.5, *τ* *=* 0.222 seconds, and *D* = 0.05 for the musician SAPPA model and *D* = 0.36 for the non-musician SAPPA model.

For all experiments that we conducted *A =* -0.5. This implies that our model’s behavior is half of the magnitude of the external stimulus exp(*i*2*π f*_*s*_ *t*), which always has a magnitude of 1, meaning that the model is forced more strongly by the external sinusoidal stimulus than by its own activity and that the SAPPA model’s phase-locking behavior will be greatly determined by the phase of the external sinusoid [50]. In the SAPPA model, all recurrent feedback terms are negative. That is why *A =* -0.5 is negative in Eqs (7) and (8), like the delayed recurrent feedback term in Eq (5). In Eq (7), *z* affects the encoding of the external stimulus, shifting the phase of *F* in a negative direction with respect to exp(*i*2*π f*_*s*_ *t*). Behaviorally, this means that when the SAPPA model listens to itself in addition to the stimulus exp(*i*2*π f*_*s*_ *t*), its actions will be more aligned with $F=\frac{exp(i2\pi \;f_{s}\;t)+Az}{\left|exp(i2\pi \;f_{s}\;t)+Az\right|}$, thus resulting in reduced anticipation compared to when *F* = exp(*i*2*π f*_*s*_ *t*).

### Experiment 2: Interpersonal synchronization during alternating paced tapping with or without auditory feedback

#### Behavioral data for simulation

Among the behavioral data shown by Nowicki and colleagues [10], we were focused on simulating the results from solo and duet tasks. First, in the solo task, musicians tapped every other beat in synchrony with a metronome while hearing their own taps (feedback-on) or only hearing the metronome (feedback-off). This means that musicians’ tapping behavior had a subharmonic rate with the stimulus rate (i.e., if the stimulus was presented at 2 Hz, tapping should occur at 1 Hz). As shown in Fig 3A, anticipation was larger when musicians could not hear their own taps compared to when they did. Second, in the duet task, pairs of musicians alternately tapped with a metronome while hearing their own and the partner’s taps (feedback-on) and also while only hearing the metronome (feedback-off). The anticipation was also larger when musicians could not hear each other in the duet task, as shown in Fig 3D.

#### Setup, procedures and measurements

To simulate the feedback-off condition, we removed the *z* term in the *F* input, meaning that the model received input only from the external sinusoid *F* = exp(*i*2*π f*_*s*_ *t*). To simulate the feedback-on condition, we added the model’s own *z* activity to the input *F* during the second half of every stimulus cycle. Finally, to simulate the duet task, we connected two SAPPA models, both synchronizing with the same external sinusoid. One of the SAPPA models exhibited in-phase synchronization with the external sinusoidal stimulus and the other one synchronized antiphase with respect to the external sinusoidal stimulus. To achieve in-phase and antiphase synchronization, the sinusoidal stimulus had a positive sign (i.e., *F* = exp(*i*2*π f*_*s*_ *t*)) and a negative sign (i.e., *F* = -exp(*i*2*π f*_*s*_ *t*)), respectively. To simulate the feedback-on condition, the two synchronizing SAPPA models alternately received one of the model’s *z* activity as input during the first half of every stimulus cycle, and the other model’s *z* activity during the second half of every stimulus cycle.

We used the SAPPA model and the parameter set determined for musicians’ data in Experiment 1. The simulations of the solo task started with a musician SAPPA model receiving an external sinusoid of frequency *f*_*s*_ = 1Hz (similarly, the SAPPA model’s *f* = 1Hz). To simulate the solo task with auditory feedback, we observed the model’s behavior while its own *z* activity was added as an additional input during the second half of every stimulus cycle (feedback-on) (see Fig 1B). This means that, the input alternates between *F =* exp(*i*2*π f*_*s*_ *t*) and *F =* exp(*i*2*π f*_*s*_ *t*) *+ Az* at a frequency twice as fast as the external sinusoid, to follow the task design (e.g., listening to a metronome click, then listening to both a metronome click and own tapping). To simulate the condition without auditory feedback, the external sinusoid was set as the only input throughout the simulation (feedback-off, the input was always *F =* exp(*i*2*π f*_*s*_ *t*)). This task design and the corresponding model setup are indicated in Fig 1B and 1C, respectively.

To simulate the duet task indicated in Fig 1D, we paired two models, of which one was referred to as model 1 and the other as model 2. To simulate the duet condition with auditory feedback, the input to model 1 was set to *F =* exp(*i*2*π f*_*s*_ *t*) *± Az(k)*, while the input to model 2 was set to *F = -*exp(*i*2*π f*_*s*_ *t*) *± Az(k)*, where |*A|* = 0.5 and *k* alternated between 1 and 2 at a rate twice as fast as the frequency of the external sinusoid, as illustrated in Fig 1D. As stated earlier, the sign of *± Az(k)* depends on whether *z* indicates recurrent feedback (positive) or the other model’s activity (negative). To simulate the duet condition without auditory feedback, both models received only the external sinusoid as input (*F* was always *F =* exp(*i*2*π f*_*s*_ *t*) for model 1 and *F = -*exp(*i*2*π f*_*s*_ *t*) for model 2). Fig 1E illustrates the simulation of the condition without auditory feedback.

The mean asynchrony between the stimulus and the in-phase synchronizing model was computed in the same manner as Experiment 1, as the difference between the peak of the real part of the models’ oscillation and the closest peak timepoints in the real part of the stimulus sinusoid. The mean asynchrony between the stimulus and the anti-phase synchronizing model was computed as the difference between the peak of the real part of the models’ oscillation and the closest valley timepoints in the real part of the stimulus sinusoid.

#### Model optimization

The SAPPA models in this experiment were the same musician and non-musician models identified in Experiment 1. Therefore, the model architecture used in this experiment is summarized by Eq (5). Simulations lasted a total of 20 seconds.

### Experiment 3: Interpersonal synchronization during rhythm-clapping alternation in the presence of transmission latencies

#### Behavioral data for simulation

In the behavioral paradigm used by Chafe and colleagues [14], two musicians in different rooms alternately clapped a staggered and looping rhythmic pattern together for an extended period of time. TLs were introduced between the musicians to simulate transmission delays over the internet when remotely located individuals play music together. Such latencies via an internet connection typically range from 20ms to 100ms [85,86]. The rhythmic task consisted of three claps interspaced with periods of relative length of 1-1-2. The two musicians synchronized and performed the pattern in a staggered manner with a half pattern overlap; the first musician started the first half of the pattern with doing two claps (1-1-) alone, and at the third clap (2-), the starting point of the second half of the pattern, the second musician started the first half of the pattern from the beginning (1-1-). When the second musician started the second half of the pattern (2-), the first musician returned to the first half of the pattern (1-1-) and so on. They repeated this without interruption for about 30 s (see Fig 1F). Before starting, the first musician heard a metronome for six counts. The metronome was randomly set at a tempo of either 86 bpm (beat-per-minute), 90 bpm, or 94 bpm to avoid habituation effects. The second musician did not hear the metronome and joined after hearing the auditory outcomes of the starter’s actions for the first half of the pattern. Delivery of auditory outcome information between subjects was bidirectionally delayed by a TL. For a given trial, the latency stayed at a constant value. Latencies between 3ms and 78ms were examined. The results show that with TLs longer than 20ms the two musicians decelerated their common tempo. For the latencies shorter than 10ms, they accelerated instead. As shown in Fig 4B, the asynchrony between the two musicians at every cycle grew as a function of the TL.

#### Setup, procedures and measurements

To simulate this paradigm, we used a pair of musician SAPPA models like the one developed in Experiment 1, and set them up to feed one model’s *z* activity as an input to the other at a given cycle, then alternate this input flow direction (see Fig 1F). The first model is considered to be the initiator and the other, the joiner. In our simulations, *f* for both oscillators was set to be a frequency of 1.5Hz, equivalent to 90 bpm. For the sake of simplicity, we did not employ the nearby offsets of this frequency that were used by Chafe and colleagues [14] to mitigate adaptation to a specific tempo by the subjects. The simulations were also carried out with pairs of non-musician SAPPA models. Compared to Experiment 1 and Experiment 2 where an external sinusoid stimulated the SAPPA model, in Experiment 3 pairs of oscillators stimulated each other, and there was no external sinusoid.

A simulation of the original experiment began by allowing the initiator SAPPA model to oscillate for one cycle. During this cycle, the initiator received its own *z* activity as an input (the input to the initiator was *F = Az*(1)) and sent its *z* activity as an input to the joiner (the input to the joiner was *F = z*(1)). After receiving an input from the initiator for one cycle, the joining model continued oscillating one cycle getting its own *z* activity as an input (the input to the joiner was *F =* -*Az*(2)) and sending its *z* activity to the initiator (the input to the initiator is then *F = z*(2)). In subsequent cycles, the inputs to the two models continued alternating every cycle which oscillator’s *z* activity was used as the inputs to both oscillators. At a given cycle, whichever model’s *z* activity was used as an input to both models was referred to as the ‘active’ model, while the other one was referred to as the ‘passive’ model. This alternation was repeated until the simulation had run for 30 seconds to complete a trial. Trials were carried out in the presence of fixed TLs ranging from 0ms to 78ms between models.

We measured the Lead/Lag relationship between models in the same way as Chafe and colleagues [14]. For each participant clapping in turn, the Lead/Lag relationship with respect to the other participant was measured as:

L=(a(1)−b(1))+(b(2)−a(2)) (9)

Where *a*(1)- *b*(1) is the time difference in seconds between the first clap of the participant currently in turn and the last clap of the participant previously in turn. *b*(2)–*a*(2) is the time difference between the first clap of the participant clapping after the current participant in turn and the last clap of the current participant in turn. *L* can be expressed as a fraction of a 90 bpm tempo, as in Chafe and colleagues [14], using the expression: - 90 x *L* / (60 x 1000*)*, where *L* (in ms) gets converted to a fraction of a period corresponding to a 90bpm tempo. If the resulting value was negative, this meant that the passive oscillator lagged behind the active oscillator. A positive value suggests the opposite.

#### Model optimization

The SAPPA model in this experiment was similar to the musician and non-musician models identified in Experiment 1. Therefore, the model architecture used in this experiment is summarized by Eq (5). Compared to Experiments 1 and 2, in this experiment these was no external sinusoidal stimulus. Instead, pairs of SAPPA models stimulated each other.

## Data and software

The software to run the simulations and generate all the figures is available in the github repository: https://github.com/iranroman/SAPPA

## Supporting information

S1 Fig The SAPPA model’s behavior when the external periodic input is a square wave instead of a sinusoid. (A) Illustration of what the asynchrony between the SAPPA model and the external square wave stimulus looks like, and how it’s measured. (B) Analysis of the asynchrony (in ms) as a function of *D* and *τ* in Eq (5) when *A* = -0.5 and *f =* 1. (C) The anticipation observed when the musician (green dots) and non-musician (yellow dots) SAPPA models were stimulated by the external square wave while also receiving their own non-delayed activity as input (*A* = -0.5). In all simulations *τ* = 0.222 seconds. The *D* parameter differentiates the musician and non-musician models. The regression lines for the behavioral data originally shown in Fig 2A are shown for comparison purposes in (C).
(DOCX)

S2 Fig Analysis of the asynchrony between the SAPPA model and the stimulus as a function of the recurrent delay *τ*. Rectangular plots (left panels) show the asynchrony as a function of *τ* (in units of seconds) for different values of *A* while *D* stays constant (*D* = 1.0; *f* = 1.0). Circular plots (right panels) show the angle of the asynchrony and the magnitude of the SAPPA model. In the rectangular plots, asynchrony is shown in units of radians, and not in seconds, in order to match the cyclic dynamic range of the circular plots. In the rectangular plots, gray-shaded areas indicate regions where the SAPPA model did not synchronize with the stimulus, and instead mode-locking was observed. Vertical dotted lines indicate values of *τ* for which circular plots were calculated. In the circular plots, individual blue lines start from different initial conditions, all of which arrive to either a red dot (a fixed point) or a red ring (a limit cycle). (A) *A* = -1.0 (B) *A* = -0.5 (C) *A* = 0.0 (D) *A* = 0.5 (E) *A* = 1.0. The limit cycle behavior is observed when the SAPPA model does not synchronize with the stimulus, and instead the SAPPA model mode-locks with the stimulus. Note: the circular plots are known as polar plots.
(DOCX)
