# Supplementary material for: Delayed feedback embedded in perception-action coordination cycles results in anticipation behavior during synchronized rhythmic action: A dynamical systems approach
Source: PLoS Comput Biol. 2019 Oct 31;15(10):e1007371. doi: 10.1371/journal.pcbi.1007371 (PMC6822724; doi:10.1371/journal.pcbi.1007371)
Supplement: S1 Fig — (A) Illustration of what the asynchrony between the SAPPA model and the external square wave stimulus looks like, and how it’s measured. (B) Analysis of the asynchrony (in ms) as a function of D and τ in Eq (5) when A = -0.5 and f = 1. (C) The anticipation observed when the musician (green dots) and non-musician (yellow dots) SAPPA models were stimulated by the external square wave while also receiving their own non-delayed activity as input (A = -0.5). In all simulations τ = 0.222 seconds. The D parameter differentiates the musician and non-musician models. The regression lines for the behavioral data originally shown in Fig 2A are shown for comparison purposes in (C). (DOCX) [file pcbi.1007371.s001.docx]

**S1 Fig. The SAPPA model’s behavior when the external periodic input is a square wave instead of a sinusoid.**  (A) Illustration of what the asynchrony between the SAPPA model and the external square wave stimulus looks like, and how it’s measured. (B) Analysis of the asynchrony (in ms) as a function of *D* and *τ* in Eq. (5) when *A* = -0.5 and *f =* 1. (C) The anticipation observed when the musician (green dots) and non-musician (yellow dots) SAPPA models were stimulated by the external square wave while also receiving their own non-delayed activity as input (*A* = -0.5). In all simulations *τ* = 0.222ms. The *D* parameter differentiates the musician and non-musician models. The regression lines for the behavioral data originally shown in Fig. 2A are shown for comparison purposes in (C).
