# Supplementary material for: Delayed feedback embedded in perception-action coordination cycles results in anticipation behavior during synchronized rhythmic action: A dynamical systems approach
Source: PLoS Comput Biol. 2019 Oct 31;15(10):e1007371. doi: 10.1371/journal.pcbi.1007371 (PMC6822724; doi:10.1371/journal.pcbi.1007371)
Supplement: S2 Fig — Rectangular plots (left panels) show the asynchrony as a function of τ (in units of seconds) for different values of A while D stays constant (D = 1.0; f = 1.0). Circular plots (right panels) show the angle of the asynchrony and the magnitude of the SAPPA model. In the rectangular plots, asynchrony is shown in units of radians, and not in seconds, in order to match the cyclic dynamic range of the circular plots. In the rectangular plots, gray-shaded areas indicate regions where the SAPPA model did not synchronize with the stimulus, and instead mode-locking was observed. Vertical dotted lines indicate values of τ for which circular plots were calculated. In the circular plots, individual blue lines start from different initial conditions, all of which arrive to either a red dot (a fixed point) or a red ring (a limit cycle). (A) A = -1.0 (B) A = -0.5 (C) A = 0.0 (D) A = 0.5 (E) A = 1.0. The limit cycle behavior is observed when the SAPPA model does not synchronize with the stimulus, and instead the SAPPA model mode-locks with the stimulus. Note: the circular plots are known as polar plots. (DOCX) [file pcbi.1007371.s002.docx]

**
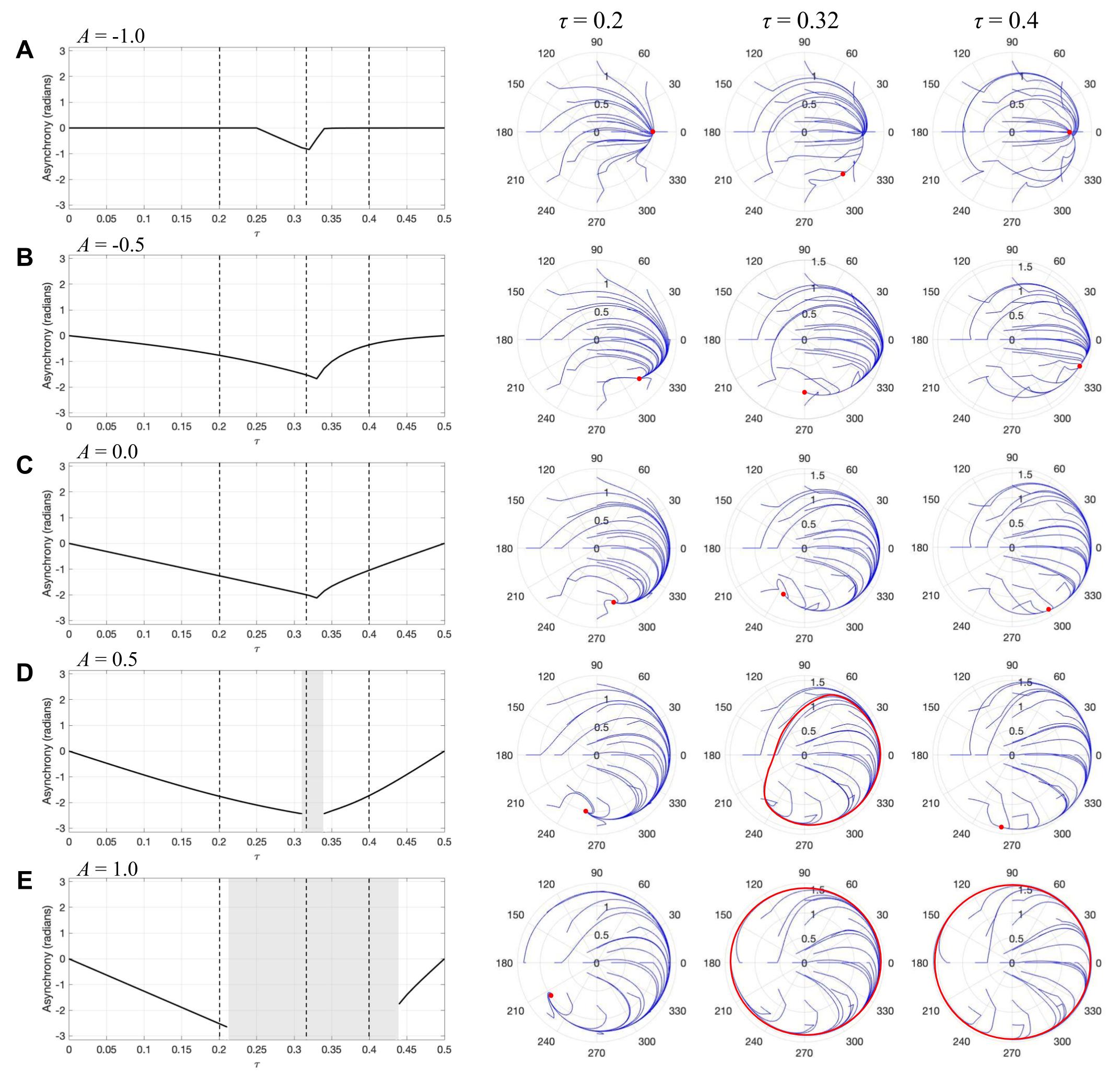
**

**S2 Fig. Analysis of the asynchrony between the SAPPA model and the stimulus as a function of the recurrent delay *τ*.** Rectangular plots (left panels) show the asynchrony as a function of *τ* (in units of seconds) for different values of *A* while *D* stays constant (*D* = 1.0; *f* = 1.0). Circular plots (right panels) show the angle of the asynchrony and the magnitude of the SAPPA model. In the rectangular plots, asynchrony is shown in units of radians, and not in seconds, in order to match the cyclic dynamic range of the circular plots. In the rectangular plots, gray-shaded areas indicate regions where the SAPPA model did not synchronize with the stimulus, and instead mode-locking was observed. Vertical dotted lines indicate values of *τ* for which circular plots were calculated. In the circular plots, individual blue lines start from different initial conditions, all of which arrive to either a red dot (a fixed point) or a red ring (a limit cycle). (A) *A* = -1.0 (B) *A* = -0.5 (C) *A* = 0.0 (D) *A* = 0.5 (E) *A* = 1.0. The limit cycle behavior is observed when the SAPPA model does not synchronize with the stimulus, and instead the SAPPA model mode-locks with the stimulus. Note: the circular plots are known as polar plots.
